# Mechanisms of Flavonoids and Their Derivatives in Endothelial Dysfunction Induced by Oxidative Stress in Diabetes

**DOI:** 10.3390/molecules29143265

**Published:** 2024-07-10

**Authors:** Baolei Dou, Yingying Zhu, Mengwei Sun, Lina Wang, Yu Tang, Shuo Tian, Furong Wang

**Affiliations:** College of Traditional Chinese Medicine, Shandong University of Traditional Chinese Medicine, Jinan 250300, China

**Keywords:** diabetic complications, flavonoids, reactive oxygen species, antioxidant enzymes, endothelial dysfunction

## Abstract

Diabetic complications pose a significant threat to life and have a negative impact on quality of life in individuals with diabetes. Among the various factors contributing to the development of these complications, endothelial dysfunction plays a key role. The main mechanism underlying endothelial dysfunction in diabetes is oxidative stress, which adversely affects the production and availability of nitric oxide (NO). Flavonoids, a group of phenolic compounds found in vegetables, fruits, and fungi, exhibit strong antioxidant and anti-inflammatory properties. Several studies have provided evidence to suggest that flavonoids have a protective effect on diabetic complications. This review focuses on the imbalance between reactive oxygen species and the antioxidant system, as well as the changes in endothelial factors in diabetes. Furthermore, we summarize the protective mechanisms of flavonoids and their derivatives on endothelial dysfunction in diabetes by alleviating oxidative stress and modulating other signaling pathways. Although several studies underline the positive influence of flavonoids and their derivatives on endothelial dysfunction induced by oxidative stress in diabetes, numerous aspects still require clarification, such as optimal consumption levels, bioavailability, and side effects. Consequently, further investigations are necessary to enhance our understanding of the therapeutic potential of flavonoids and their derivatives in the treatment of diabetic complications.

## 1. Introduction

Diabetes has become a widespread problem of epidemic proportions. As reported by the International Diabetes Federation, the number of individuals aged 20–79 years with diabetes worldwide was 537 million in 2021. Moreover, the incidence rate of diabetes is rapidly increasing, and it is estimated to reach 783 million by 2045 [1]. Diabetic complications are the leading cause of mortality among diabetic patients and significantly impact their quality of life. They are divided into macrovascular complications and microvascular complications in the clinic. Hyperglycemia (HG)-induced endothelial dysfunction is the key to vasculopathy. The endothelium is a continuous monolayer of cells that lines the arterial, venous, and lymphatic vessels. It constantly faces the force of blood pressure and shear flow [2,3]. Endothelial dysfunction can be narrowly defined as a decrease in the capacity for vasodilation. However, it can also encompass any alteration that influences the endothelium’s foundational function for vascular protection. This includes the impairment in vascular tone, compromised integrity of the endothelium, malfunctioning endothelial cell metabolism, and platelet activation [4]. Reactive oxygen species (ROS), which act as intermediates of molecular oxygen (O_2_), are important secondary messengers of cell metabolism. However, oxidative stress (OS), which is an imbalance between antioxidant enzyme defense systems and ROS generation, is a crucial factor in endothelial dysfunction in diabetes [5]. Excess ROS induces inflammation, a reduction in nitric oxide (NO), and mitochondrial dysfunction in endothelial cells (ECs) [6]; additionally, inflammation enhances ROS generation in turn [7].

Flavonoids, which consist of 15 carbon skeletons and two aromatic rings connected by three carbon chains, are phenolic compounds [8]. Accumulating evidence strongly suggests that flavonoids inhibit vascular injury in diabetes [9]. Pycnogenol, as a flavonoid-rich dietary supplement, has been shown, in a clinical study of patients with type II diabetes mellitus, to significantly decrease endothelin-1 (ET-1) levels in serum and reduce the dosages of the vasoconstrictor angiotensin-converting enzyme (ACE) inhibitor in the treatment group [10]. The antioxidant properties of flavonoids are the key to their protective effect and are closely associated with their structural characteristics; they are related to conjugated double bonds, the position and number of hydroxyl groups, and electrophilic groups in these rings [11]. In this review, we first describe the generation of OS in diabetes. Subsequently, we present a summary of the findings on the mechanisms mediated by flavonoids against endothelial dysfunction in diabetes.

In preparing this review, we searched related articles (as of 2 July 2024) from the PubMed database. The review includes two main sections, and the search terms of oxidative stress and endothelial dysfunction in diabetes were (“Diabetes” [Mesh] OR Diabatic [Title/Abstract] OR Hyperglycemia [Title/Abstract]) AND (“Oxidative Stress” [Mesh] OR Reactive Oxygen Species [Title/Abstract] OR Reactive Nitrogen Species [Title/Abstract] OR Antioxidative [Title/Abstract]) AND (“Endothelial Dysfunction” [Mesh] OR Endothelial [Title/Abstract] OR Endothelium [Title/Abstract]). The search terms of flavonoids were (“Flavonoids” [Mesh] OR Flavonoid Derivatives [Title/Abstract]) AND (“Diabetic Complications” [Mesh] OR Hyperglycemia [Title/Abstract] OR Diabetes) AND (“Endothelial Dysfunction” [Mesh] OR Endothelial [Title/Abstract] OR Endothelium [Title/Abstract]). To focus on endothelial function, we excluded articles with non-endothelial cell samples in the cellular experiments, and among the screened articles with animal experiments alone, we retained only those that examined endothelial function in a narrow sense, including vasodilation, NOS activity, and NO bioavailability. Finally, 167 articles were included in this review.

## 2. The Generation of Diabetes-Induced Oxidative Stress in Endothelial Cells

Superoxide anions (O_2_**^·^**^−^) and hydrogen peroxide (H_2_O_2_), which participate in O_2_ metabolites, are the main ROS. Moreover, other ROS include hydroxyl radicals (OH^·^), hydroxyl anions (OH^−^), and hypochlorous acid (HOCl). In diabetes, the sources of ROS include the mitochondrial electron transport chain (ETC), nicotinamide adenine dinucleotide phosphate (NADPH) oxidase, xanthine oxidase (XO), and uncoupled endothelial nitric oxide synthase (eNOS). Moreover, uncoupled eNOS forms highly reactive peroxynitrite (ONOO^−^) [7].

### 2.1. Mitochondrial-Derived Reactive Oxygen Species in Diabetes

The mitochondrial metabolism plays a crucial role in EC growth, apoptosis, proliferation, and mobilization [12]. In a clinical study, mitochondrial fission was found to be increased in diabetic patients and led to endothelial dysfunction [13]. In addition, mitochondria serve as a significant source of ROS. NADH-ubiquinone oxidoreductase (complex I) and ubiquinol-cytochrome C reductase (complex III) predominantly contribute to O_2_**^·^**^−^ generation within the electron transport chain (ETC) [14].

Under pathophysiological conditions associated with diabetes, mitochondrial dysfunction promotes the generation of ROS, disrupts the endothelial NO metabolism, inhibits proliferation, and stimulates apoptosis, which are all closely associated with endothelial dysfunction development in diabetes [15]. In ECs, HG suppresses the activity of the tricarboxylic acid cycle and ETC, which are sources of mitochondrial ROS [16]. Keller and colleagues showed that glycated low-density lipoprotein (glyLDL) inhibited the activity of mitochondrial ETC enzymes [17], which is consistent with the results that revealed that HG suppressed mitochondrial electron transport. Moreover, other studies showed that diabetes-associated metabolic disorders decreased oxygen consumption, mitochondrial membrane potential, cell viability, and enzymatic activity in ECs cultured with HG [18,19,20]. Subsequently, HG induced intramitochondrial O_2_**^·^**^−^ production, leading to the activation of the amino hexose signaling pathway, polyol signaling pathway, protein kinase C (PKC) signaling pathway, and advanced glycosylation end-products (AGEs); ultimately, these processes led to endothelial dysfunction [21].

### 2.2. NADPH Oxidase-Derived Reactive Oxygen Species in Diabetes

All NADPH oxidase (NOX) family members, which are membrane-bound enzymes, are acknowledged as the primary sources of ROS in diabetes [22]. NOX1, NOX2, NOX4, and NOX5 have emerged as the prominent contributors of OS in ECs. Studies have shown that NOX are activated by the upregulation of their subunit p22^phox^ and p47^phox^ translocation in diabetes [23].

Several studies have provided abundant evidence regarding the excessive production of NOX-derived ROS in diabetic models. In human aortic endothelial cells (HAECs) stimulated with HG, the overexpression of NOX2, NOX4, and p47^phox^ is induced by HG [24]. In mouse brain microvascular endothelial cells treated with HG, HG enhanced the activity of NOX by upregulating the expression of the NOX1 protein. Subsequently, resveratrol, which is a kind of nuclear factor-kappa B (NF-κB) inhibitor, prevented the upregulation of NOX1 expression [25]. This demonstrated that the activation of NF-κB-mediated NOX1 is an important process of OS in diabetes. Moreover, HG increased the generation of NOX4-mediated ROS in HAECs. The interaction between NOX4 and NF-κB/p65 was enhanced by HG. Subsequently, an intervention with NF-κB/p65 inhibition or the knockdown of NF-κB/p65 via small interfering RNA (siRNA) prevented the overexpression of NOX4 induced by HG [26]. Hence, this study showed the mechanism of NF-κB/p65-mediated NOX4 overexpression in HG conditions. In addition, evidence has shown that the AMP-activated protein kinase (AMPK)/PKC pathway is also responsive to NOX expression. In human umbilical vein endothelial cells (HUVECs) stimulated with different concentrations of glucose (5 and 10 mmol/L), rosiglitazone inhibited NOX-mediated OS via AMPK signaling pathways, which subsequently inhibited PKC and the translocation of the NOX subunits p47^phox^ and Ras-related C3 botulinum toxin substrate 1 [27]. Similarly, the expression of gp91^phox^, the catalytic subunit of NOX2, increased with HG in porcine coronary rings, and the increase in O_2_**^·^**^−^ was completely blocked by staurosporine’s PKC inhibition [28], which indirectly predicted that the PKC pathway was responsive to NOX2. The same result was verified in another experiment of HUVECs treated with intermittent and continued HG, and PKC inhibition reduced the increase in p47^phox^, p67^phox^, and p22^phox^ induced by HG [29]. Recently, a study of NOX5 transgenic mice demonstrated the HG-induced upregulation of NOX5 expression in vascular ECs and smooth muscle cells [30]. Furthermore, studies have indicated that NOX5 activation is related to PKC pathways and that PKCα mediates NOX5 activation in HUVECs exposed to HG [31]. In summary, the evidence of endothelial cells exposed to the HG environment indicates an important role of NOX-derived ROS in stimulating endothelial cells’ OS.

There is also considerable evidence that correlates vascular complications with NOX overexpression in diabetes. The aorta of streptozotocin (STZ)-induced diabetic rats exhibited an increase in NOX1 protein and a reduction in endothelium-dependent vasodilation. However, it was difficult to prove that NOX1 is the prominent source of endothelial dysfunction because there were other concurrent observations, such as eNOS uncoupling and xanthine oxidase overexpression [32]. In another study, Youn et al. interfered with the siRNA transfection mixture of NOX1, NADPH oxidase organizer 1, NOX4, and mitochondrial ETC complex III in diabetic mice, and the results suggested that NOX1 is an important contributor to eNOS uncoupling and the subsequent occurrence of endothelial dysfunction [33]. Other studies highlighted NOX2-induced endothelial impairments in animal models of diabetes. Sukumar et al. created mouse models with endothelial-specific insulin resistance to show impairments in endothelium-dependent vasodilation, the overproduction of superoxide, and overexpression of NOX2 in pulmonary ECs isolated from the aorta and lungs of transgenic mice; subsequently, OS and vascular endothelial dysfunction were inhibited by the knockdown of siRNA against NOX2 [34]. NOX4, which generates H_2_O_2_, is the major isoform in ECs. However, the protective effect of NOX4 on endothelial function is controversial. Craige et al. demonstrated that NOX4 promoted endothelial angiogenesis through eNOS activation in hypoxia and ischemia models. In contrast, Wang et al. showed that HG induced a decrease in NO bioavailability via the overexpression of NOX4 in the aorta of diabetic rats [35]. To date, there have been few rescue experiments on endothelial function by NOX4 in diabetic models; so, it is worth exploring the mechanisms of NOX4 on vascular function.

### 2.3. Uncoupled eNOS-Derived Reactive Oxygen Species in Diabetes

Nitric oxide synthases are enzymes that catalyze the production of nitric oxide (NO) from L-arginine, and the cofactor tetrahydrobiopterin (BH4) [4], including the three isoforms eNOS and neuronal nitric oxide synthase (nNOS) (which are expressed under normal conditions) and inducible nitric oxide synthase (iNOS), is induced by impairment.

The generation of NO is a compact process in the eNOS enzyme. However, metabolic disorders induce eNOS uncoupling in diabetes, which leads to reverse electron transfer; the process generates O_2_**^·^**^−^ and no longer synthesizes NO [36,37]. Moreover, O_2_**^·^**^−^ reacts with NO to generate ONOO^−^, which depletes NO, decreases the bioavailability of NO, and then impairs endothelial function [38]. In a rat model of diabetes, observations of the aorta showed the occurrence of oxidative stress and a decrease in NO [39]. Furthermore, the function of NO-mediated vasodilation was significantly impaired [40,41].

### 2.4. Reactive Oxygen Species Derived from other Enzymes in Diabetes

Xanthine oxidoreductase (XOR), an enzyme (270 kDa) involved in the metabolic process of purines, facilitates the oxidation of hypoxanthine, resulting in the production of xanthine and eventually uric acid. XOR includes two forms, xanthine oxidase and xanthine dehydrogenase, and XO is the main superoxide-producing enzyme [42]. In a clinical trial on the correlation between XO and diabetes mellitus, the mean XO level of the diabetes group (5.8 ± 3.6 U/L) was significantly higher than that of the control (2.9 ± 1.8 U/L) group [43]. Studies have demonstrated that XOR activity is related to endothelial dysfunction in diabetes [44,45], which may be due to ROS produced by XOR attenuating NO-mediated vasorelaxation. In a randomized, controlled trial of diabetes and mild hypertension, allopurinol, a xanthine oxidase inhibitor, improved the response of acetylcholine (Ach)-mediated blood flow and decreased malondialdehyde (MDA) levels [46]. In addition, allopurinol decreased the level of xanthine-dependent superoxide in the aorta of diabetic animals [47]. These data indicate that XO-derived superoxide is one of the factors of endothelial dysfunction in diabetes.

**Figure 1:**
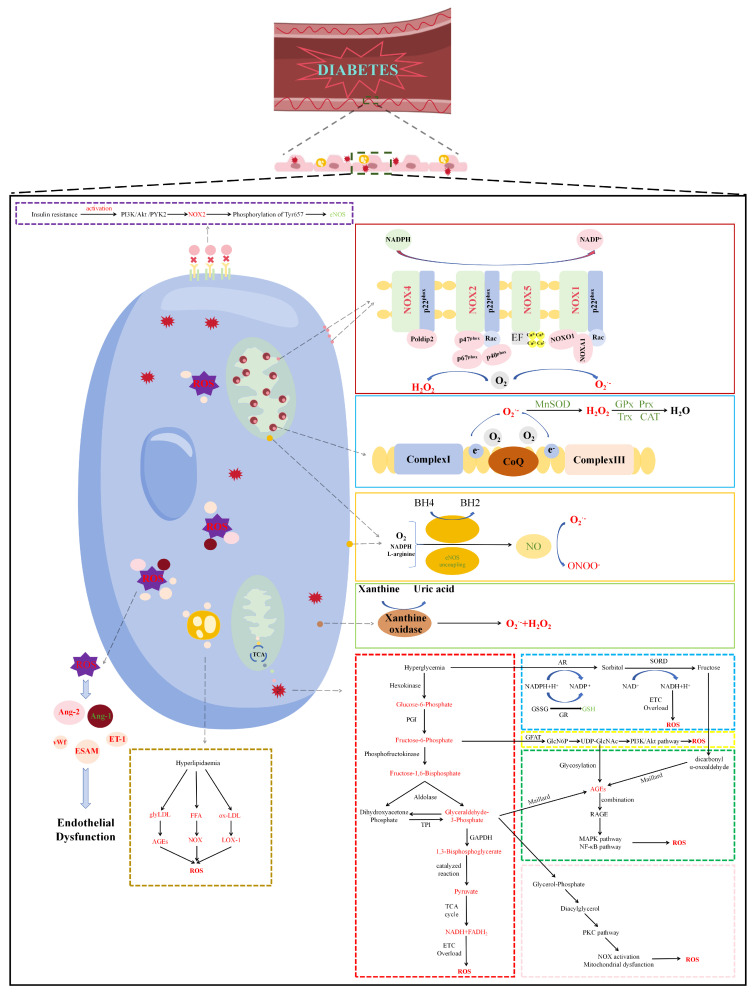
Mechanisms of oxidative stress-induced endothelial dysfunction in diabetes. In the diabetic state, oxidative stress induces endothelial dysfunction via the regulation of endothelium-related factors, including Ang-2, vWf, ESAM, ET-1, and Ang-1. Specifically, the dark red solid frame shows ROS generated by NOX family in diabetes, including NOX1, NOX2, NOX4, and NOX5. The light blue solid frame shows ROS generated by the leakage of electrons in the mitochondrial ETC and decrease in antioxidant enzyme activity. The orange solid frame shows that eNOS uncoupling leads to a decrease in NO and generates ONOO^−^ in diabetes. The light green solid frame shows ROS generated by xanthine oxidase in diabetes. The red dashed frame shows that hyperglycemia accelerates glycolysis to generate excess pyruvate, then leading to mitochondrial overload. The light blue dashed frame shows ROS generated by the polyol pathway in diabetes. The yellow dashed frame shows ROS generated by the hexosamine pathway via the acceleration of glycation in diabetes. The green dashed frame shows that the binding of AGEs and RAGE activates MAPK and NF-κB pathways to generate ROS. The scarlet dashed frame shows the process of ROS generated via the PKC pathway in diabetes. The brown dashed frame shows ROS generated by glyLDL, FFA, and ox-LDL in diabetes. The purple dashed frame shows eNOS uncoupling induced by insulin resistance in diabetes. Abbreviations: PI3K, phosphatidylinositol 3-kinase; Akt, protein kinase B; PYK2, proline-rich tyrosine kinase 2; NOX, NADPH oxidase; eNOS, endothelial nitric oxide synthase; ROS, reactive oxygen species; TCA cycle, tricarboxylic acid cycle; Ang-2 angiopoietin-2; vWf, von Willebrand factor; ESAM, endothelial cell-selective adhesion molecule; ET-1, endothelin-1; Ang-1, angiopoietin-1; glyLDL, glycated low-density lipoprotein; FFA, free fatty acid; ox-LDL, oxidized low-density lipoproteins; AGEs, advanced glycosylation end-products; LOX-1, lectin-like ox-LDL receptor-1; NADPH, nicotinamide adenine dinucleotide phosphate; EF, EF hand; NOXO1, NADPH oxidase organizer 1; NOXA1, NADPH oxidase activator 1; Rac, Ras-related C3 botulinum toxin substrate; MnSOD, manganese superoxide dismutase; GPx, glutathione peroxidase; Prx, peroxiredoxin; Trx, thioredoxin; CAT, catalase; CoQ, coenzyme Q; BH4, tetrahydrobiopterin; BH2, dihydrobiopterin; O_2_, molecular oxygen; O_2_^−^, superoxide; ONOO^−^, peroxynitrite; H_2_O_2_, hydrogen peroxide; PGI, phosphoglucose isomerase; TPI, triosephosphate isomerase; GAPDH, glyceraldehyde-3-phosphate dehydrogenase; FADH_2_, flavin adenine dinucleotide; ETC, electron transport chain; AR, aldose reductase; SORD, sorbitol dehydrogenase; GSSG, glutathione disulfide; GR, glutathione reductase; GFAT, glutamine-fructose-6-phophate amidotransferase; RAGE, receptor for advanced glycation end-products; MAPK, mitogen-activated protein kinase; NF-κB, nuclear factor-kappa B; PKC, protein kinase C.

Excess superoxide leads to the release of Fe^2+^ by ferritin and iron–sulfur cluster-containing proteins. The increased Fe^2+^ produces highly reactive OH^−^ and OH^·^, which is referred to as Fenton’s reaction or ferroptosis [7]. Recent research has shown that ferroptosis contributes significantly to the development of diabetes and its complications [48]. In HUVECs stimulated with HG and interleukin 1β (IL-1β), Luo and colleagues treated cells with deferoxamine and ferrostatin-1, demonstrating that ferroptosis inhibitors reduce lipid peroxides and improve endothelial cell viability. Mechanistically, HG and IL-1β enhanced HUVEC ferroptosis by activating the p53/xCT/GSH pathways. Moreover, a reduction in xCT and endothelial cell content was observed in the aorta of diabetic mice [49]. The main sources of ROS are depicted in Figure 1.

### 2.5. Attenuation of Antioxidant Defense Systems in Diabetes

To preserve the redox balance, the presence of antioxidant defense mechanisms, which function to eliminate ROS, is vital. The antioxidant enzymes that actively participate in this process include superoxide dismutase (SOD), glutathione peroxidase (GPx), catalase (CAT), peroxiredoxins (Prx), and thioredoxin (Trx) [14]. In the redox process, SOD facilitates the dismutation of O_2_^·−^ to H_2_O_2_. Subsequently, H_2_O_2_ undergoes catalysis to form H_2_O, with the assistance of CAT, GPx, Prx, and Trx, through a reduction reaction. As shown in Figure 1, HG inhibits the activity of antioxidant enzymes.

Superoxide dismutase (SOD) enzymes are essential in maintaining cellular homeostasis by catalyzing the dismutation of superoxide radicals into molecular oxygen and hydrogen peroxide. There are three different isoforms of SOD that include copper–zinc superoxide dismutase (CuZnSOD or SOD1, 32 kDa) located in the cytosol and nucleus, manganese superoxide dismutase (MnSOD or SOD2, 93 kDa), and extracellular CuZnSOD (ECSOD or SOD3). In the aorta of STZ/nicotinamide-induced diabetic mice, Taguchi et al. observed a decrease in SOD1 expression. However, treatment with a Ginkgo biloba extract elevated SOD1 expression and improved endothelium-dependent relaxation by activating the protein kinase B (Akt)/eNOS pathways [50]. In addition, the inhibition of SOD1 inhibited angiogenesis by increasing superoxide levels and reducing vascular endothelial growth factor (VEGF) and fibroblast growth factor-2 (FGF-2)-mediated extracellular signal-regulated kinase (Erk) 1/2 phosphorylation in ECs [51]. Moreover, H_2_O_2_ generated from SOD1 has the effect of an endothelium-dependent hyperpolarization factor [52]. Similarly, SOD2-derived H_2_O_2_ affects the formation of sprouts and blood vessel formation [53]. Increases in superoxide levels and impairment in Ach-mediated vasodilation were shown in the thoracic aorta of diabetic mice with SOD2 deficiency [54]. In vitro, the expression of SOD2 protein and SOD activity were inhibited by HG in HUVECs, and ROS-derived SOD decreased cell viability [55].

Catalase is a cytoplasmic 240 kDa homotetrameric enzyme. The decrease in CAT activity is considered to be a cause of OS in diabetes. In the aorta of diabetic rats, the observations were excess ROS and vascular pathological damage, with a decrease in CAT levels [39]. Leff et al. employed human serum catalase to protect endothelial cell function from H_2_O_2_ [56]; presumably, CAT inhibited endothelial dysfunction via the reduction of H_2_O_2_ in diabetes.

Glutathione peroxidase, an 85 kDa protein dependent on selenium, demonstrates a comparable antioxidant impact on H_2_O_2_ akin to that of catalase. Both enzymes function in the detoxification of H_2_O_2_ by transforming monomeric glutathione (GSH) into dimeric glutathione disulfide (GSSG). The family includes four different GPxs (GPx1–4) in mammals [57]. HG inhibits GSH and GPx levels in endothelial cells [58,59,60]. Recently, a reduction in GSH was reported in diabetes, indicating an important role in endothelial dysfunction via the acceleration of OS [61].

Peroxiredoxins, which reduce H_2_O_2_ to H_2_O by active cysteine residues, are a family of antioxidant enzymes. Currently, six different peroxiredoxins (Prx1–6) have been identified [7]. In a controlled clinical trial of diabetes with peripheral atherosclerosis, the levels of Prx1, Prx2, Prx4, and Prx6 were higher than those in the control group [62]. Notably, peroxiredoxins have a positive effect in protecting against inflammation and OS in cardiovascular diseases [63]. Presumably, the overexpression of Prx1, Prx2, Prx4, and Prx6 provided compensatory protection against diabetic cardiovascular issues.

Thioredoxins, which reduce disulfides to their corresponding sulfhydryls via the reduction of equivalent NADPH, are a family of 10–12 kDa redox-sensitive antioxidant enzymes located in the cytosol and mitochondria [64]. Currently, three Trx enzymes (Trx1, 2, and 3) have been identified. The positive effects of the thioredoxin family, which consists of Trx, NADPH, are regarded as antioxidant defense systems in diabetes and cardiovascular disease, and thioredoxin reductase [65]. Currently, Trx-interacting proteins have been discovered, including p40^phox^ and vitamin D3-upregulated protein 1; p40^phox^ is an NADPH subunit. The interaction indicated that Trxs might also regulate superoxide via NOXs [7]. In ECs, the expression of the Trx protein is regulated by stimulation with H_2_O_2_, and lower concentrations of H_2_O_2_ enhance Trx levels and improve cell apoptosis, while at higher concentrations, the reduction in Trx protein induces cell apoptosis in a cathepsin-D-dependent manner [66]. Thioredoxin-interacting proteins (TXNIPs) are a key factor in glucose homeostasis, and Li et al. indicated that endothelial dysfunction is positively correlated with TXNIP concentrations in diabetic rats [67]. Additionally, the knockdown of TXNIP reduced diabetes ischemia-related impairment in angiogenesis and blood flow [68]. Lam et al. generated TXNIP knockout and TXNIP overexpression mouse models and observed that the impairment in Ach-mediated vasorelaxation and the increase in nucleotide-binding domain-like receptor 3 (NLRP3) levels induced by diabetes were attenuated in the TXNIP knockout model. Additionally, the reductions in Akt phosphorylation (p-Akt) and p-eNOS were also restored [69].

## 3. Mechanisms of Oxidative Stress-Induced Endothelial Dysfunction in Diabetes

Endothelin-1, angiopoietin-1 (Ang-1), angiopoietin-2 (Ang-2), von Willebrand factor (vWf), endothelial cell-selective adhesion molecule (ESAM), and other cell adhesion molecules play a crucial role in regulating endothelial cell function through multidimensional interactions. ET-1, a vasoconstrictor peptide, inhibits eNOS activity and NO production. It has been observed that ET-1 levels are upregulated in the plasma of diabetic mice [70]. Moreover, the upregulation of ET-1 has been found to accelerate the progression of atherosclerosis, perivascular OS, and inflammation through NOX1 in diabetes [71]. Additionally, Binjawhar et al. revealed HG-induced ET-1 mRNA overexpression rather than promoter methylation in HUVECs [72]. Ang-1 activates the EC tyrosine kinase receptor TIE-2, promoting vascular integrity. It is an endothelium-specific protective factor. On the other hand, Ang-2 is involved in cardiovascular remodeling, which involves altering the vascular structure [73,74]. HG and AGEs inhibit p-Akt and p-FOXO1 induced by Ang-1 and enhance Ang-2 generation. This process is accompanied by OS and cell proliferation impairment [75]. vWf, an adhesive glycoprotein expressed in ECs, plays a crucial role in platelet adhesion and aggregation, thereby regulating vascular hemostasis. In the context of endothelial dysfunction, the plasma levels of vWf are significantly elevated in individuals with diabetes and cardiovascular diseases compared to those with diabetes alone [76]. The increase in ROS production accelerated vWf multimers to form endothelial Weibel–Palade bodies in diabetes, and then promoted vWf multimers to be released into the plasma [77]. ESAM expression was upregulated in response to HG conditions, resulting in cell adhesion and infiltration [78,79]. Additionally, other endothelial cell adhesion molecules, including E-selectin, vascular cell adhesion molecule-1 (VCAM-1), and intercellular adhesion molecule-1 (ICAM-1), were upregulated in diabetes, and their overexpression was positively correlated with OS [80]. Furthermore, in diabetes, there is an upregulation of other adhesion molecules on endothelial cells, such as vascular cell adhesion molecule-1 (VCAM-1), intercellular adhesion molecule-1 (ICAM-1), and E-selectin. This upregulation is positively correlated with oxidative stress [79]. It is crucial to inhibit endothelial injury to prevent vascular complications in diabetes. HG-induced OS has a negative impact on these factors that are related to endothelial function.

## 4. The Mechanism of Flavonoids and Their Derivatives in Endothelial Dysfunction in Diabetes

Flavonoids and their derivatives have shown positive effects in the treatment of diabetic complications. They play a protective role by modulating multiple factors. Detailed information is shown in Table 1.

### 4.1. Flavonols

Flavonols are characterized by an unsaturated carbon ring at C2-C3 as well as oxidation at C4 and hydroxylation at C3. They exist abundantly in capers, parsley, saffron, dill weed, and elderberry [115].

#### 4.1.1. Quercetin

Quercetin (C_15_H_10_O_7_) or 3,3′,4′,5,7-pentahydroxyflavone is a flavonoid compound with the highest intake in daily diets. It exists abundantly in tea, tomatoes, onions, broccoli, and cabbage [116]. Quercetin showed a positive effect on improving endothelial dysfunction in diabetes [61]. In the aorta of STZ-induced diabetic mice, quercetin promoted Ach-mediated vasodilation and eNOS-mediated NO generation via the activation of phosphatidylinositol 3-kinase (PI3K)/Akt and AMPK signaling pathways [41]. In HUVECs exposed to HG, quercetin inhibited OS and reversed the HG-induced attenuation of cell viability, autophagy, proliferation, and migration [58]. Additionally, quercetin protected NAD^+^ and redox status from HG by inhibiting the activation of poly(ADP-ribose)-polymerase (PARP) and aldose reductase (AR) in HUVECs [117]. In myeloperoxidase (MPO) and HG-cultured HUVECs, quercetin not only inhibited the generation of HOCL by directly binding to the active heme of MPO but also reduced the production of H_2_O_2_ by inhibiting NOX4 overexpression [118]. In endothelial progenitor cells (EPCs), quercetin attenuated HG-induced impairment in cell viability, OS, and cyclic 3′,5′-guanosine monophosphate (cGMP) levels and improved NO levels by inducing Sirtuin 1 (SIRT1)-dependent p-eNOS [119]. In vivo, treatment with quercetin and exercise decreased vascular injury via an increase in antioxidant enzyme activity and a decrease in OS production and iNOS levels in the aorta of diabetic models [39].

#### 4.1.2. Hyperoside

Hyperoside (C_21_H_20_O_12_) or quercetin 3-O-β-D-galactopyranoside is a flavonol extracted from Crataegus and Hypericum monogynum. It is based on the backbone of quercetin with a D-galactoside at C3. Hyperoside showed a protective role in diabetic retinopathy. In STZ and high-fat diet (HFD)-induced diabetic retinopathy model rats, treatment with hyperoside reduced retina pathological damage, cell apoptosis, and impairment in cell viability and proliferation. In vitro, hyperoside protected rat retinal vascular endothelial cells (RVECs) from apoptosis and OS induced by HG. The observations were a reduction in cysteinyl aspartate specific proteinase-3 (caspase-3), caspase-9, Bcl-2-associated X (Bax), and CytC levels and increase in B-cell lymphoma 2 (Bcl-2) [81].

#### 4.1.3. Icariin

Icariin (C_33_H_40_O_15_) is a glycosyloxyflavone obtained from the genus Epimedium. It is based on kaempferol, which is replaced with a 3-methylbut-2-en-1-yl group at A8 and in which the hydroxy groups are replaced with 6-deoxy-α-L-mannopyranoside at C3, methyl ether at B4′, and β-D-glucopyranoside at A7. Icariin showed a positive effect on inhibiting OS, inflammation, cell apoptosis, and adhesion in HUVECs exposed to HG [82]. Icariin reserved the phenylephrine (PE)-induced vasoconstriction and Ach-induced vasodilation in the aortas of alloxan-induced diabetic mice. Mechanistically, it reduced HG-induced endothelial dysfunction by inhibiting p-p38 MAPK overexpression and activating p-eNOS in HUVECs [120]. In EPCs treated with H_2_O_2_, icariin might reduce apoptosis and autophagy via the recovery of mTOR/p70S6K/4EBP1 pathway phosphorylation and the inhibition of Erk1/2 and ATF2 overexpression [121].

Icariside II is a secondary product of icariin. In the penis of diabetic rats, icariside II improved α-smooth muscle actin, nNOS, and eNOS levels and reduced the apoptosis of cavernous smooth muscle cells and ECs by inhibiting transforming growth factor β1 (TGFβ1)/drosophila mothers against decapentaplegic protein 2 (Smad2)/connective tissue growth factor (CTGF) pathways [83], and icariside II inhibited OS by restoring SOD activity and reducing lipid peroxidation (LPO) [84]. In human cavernous endothelial cells treated with HG, icariside II prevented endothelial cell dysfunction via the recovery of total antioxidant capacity (TAC), proliferation, p-Akt, p-Erk1/2, and p-eNOS [122]. Furthermore, in the kidney cortex and medulla of STZ-induced diabetes, icariside II ameliorated diabetic nephropathy via an increase in endothelial cell contents and the downregulation of the TGFβ1/Smad2/CTGF signaling pathways [123].

#### 4.1.4. Myricetin

Myricetin (C_15_H_10_O_8_) is hexahydroxyflavone in which flavonol is replaced with hydroxy groups at C3, B3′, B4′, A5, B5′, and A7. It is found in the leaves of Myrica rubra. Generally, exposure to HG causes a decrease in cell viability, and Aminzadeh et al. showed that OS and cell apoptosis were the key mechanisms of endothelial function in HUVECs exposed to HG. These observations were increased in LPO and proapoptotic protein and decreased in TAC and total thiol molecules. Additionally, treatment with myricetin reversed these trends. These results suggest that myricetin has the potential to prevent the development of endothelial dysfunction in diabetes [85].

#### 4.1.5. Rutin

Rutin (C_27_H_30_O_16_), which is based on quercetin and hydroxy group substituted by glucose and rhamnose sugar groups at C3, is a rutinoside. In vivo, rutin inhibited endothelial dysfunction. Wang et al. constructed rat models of high glucose diet feeds, and they indicated that rutin restored PE-mediated vasoconstriction and Ach-mediated vasodilation in the aorta. In vitro, rutin improved HUVEC viability and attenuated HG-induced OS-related inflammation [86], and it also improved NAD^+^ levels via the inhibition of PARP activation and AR activity [117]. In addition, rutin protected renal endothelial barrier function from HG via the inhibition of nuclear factor-E2-related factor 2 (Nrf2)-mediated ROS/Ras homolog family member A/Rho-associated coiled-coil kinase signaling pathways [124].

#### 4.1.6. Fisetin

Fisetin (C_15_H_10_O_6_) is a tetrahydroxyflavone with additional hydroxy groups at C3, B3′, B4′, and A7. The vascular endothelium exposed to HG conditions elicited an increase in permeability, monocyte–endothelial adhesion, inflammation, and OS. Treatment with fisetin ameliorated this damage; it inhibited the activation of NF-κB and decreased the overexpression of CAM, monocyte chemotactic protein-1 (MCP-1), and IL-8 [87]. Additionally, fisetin inhibited endothelial dysfunction induced by oxidized LDL via the upregulation of Erk5/myocyte-specific enhancer factor 2C/Krüppel-like factor 2 signaling pathways [88].

#### 4.1.7. Morin

Morin (C_15_H_10_O_7_) is a pentahydroxyflavone in which flavonol is substituted by hydroxy groups at B2′, C3, B4′, A5, and A7. It is found in Lotus ucrainicus and Psidium guajava. In the aorta of diabetic rats induced by STZ, morin improved NO-mediated vasodilation by activating p-Akt and p-eNOS [40,41].

#### 4.1.8. 3′,4′Dihydroxyflavonol

3′,4′-Dihydroxyflavonol (C_15_H_10_O_5_, DiOHF) is not a naturally occurring flavonoid. DiOHF has antioxidation capacity and improve Ach-mediated vasodilation in the aortic tissues of diabetic rats [125]. In diabetic rat mesenteric arteries, DiOHF reduced superoxide levels by inhibiting NOX2 overexpression and improved Ach and NO-mediated endothelium-dependent vasodilation by inhibiting eNOS uncoupling [89,126].

### 4.2. Isoflavones

Isoflavones, which are found in soybeans, microbes, and other leguminous plants, are a distinct subclass of flavonoids.

#### 4.2.1. Daidzein

Daidzein (C_15_H_10_O_4_) is a phytoestrogen that is mainly found in nuts, fruits, and soybeans. It is an isoflavone replaced with two additional hydroxy groups at B4′ and A7. Daidzein improved endothelial function and OS in diabetic rat models; it inhibited the increase in PE-mediated endothelium-dependent vasoconstriction and improved Ach-induced relaxation in aortic rings [127]. In vitro, daidzein attenuated HG-induced OS, cell viability damage, and activation of NF-κB [59]. Moreover, the exposure of HUVECs to HG and H_2_O_2_ conditions induced cell apoptosis and proliferation damage, and treatment with daidzein reversed these observations via an increase in estrogen receptor β expression and Bcl-2/Bax as well as the modulation of PI3K signaling pathways [128].

#### 4.2.2. Puerarin

Puerarin (C_21_H_20_O_9_) is an isoflavone replaced with two hydroxy groups at B4′ and A7, as well as a β-D-glucopyranosyl residue at A8 via C-glycosidic linkage. In mouse vascular endothelial exposed to HG conditions, puerarin attenuated cell permeability and NLRP3-related factors via the inhibition of OS [90].

#### 4.2.3. Calycosin-7-O-β-D-Glucopyranoside

Calycosin-7-O-β-D-Glucopyranoside (C_22_H_22_O_10_, C7G) is found in Astragalus mongholicus and Maackia amurensis. C7G is calycosin replaced with a β-D-glucopyranosyl residue at A7 via a glycosidic linkage. In HUVECs stimulated with AGEs, the Bad/Bcl-2 and MDA/SOD ratios were increased, and IL-6, ICAM-1, TGFβ1, and MCP-1 levels were increased. Treatment with C7G reversed these increases and receptor for AGEs (RAGE) levels, indicating that C7G ameliorated AGE-induced OS, inflammation, cell apoptosis, migration, and adhesion. Furthermore, the protective effect might be based on the inhibition of NF-kB and p-Erk1/2 [91].

#### 4.2.4. Kakkalide

Kakkalide (C_28_H_32_O_15_) is found in Viola hondoensis and Pueraria montana. Palmitic acid, a dominant saturated free fatty acid, plays a crucial role in the development of insulin resistance in individuals with diabetes. In a study conducted on HUVECs, the presence of kakkalide effectively suppressed the generation of ROS induced by palmitate. Additionally, it enhanced the mitochondrial membrane potential and curtailed ROS-related inflammation. Furthermore, kakkalide positively modulated insulin receptor substrate-1 function, thus improving the PI3K/Akt/eNOS pathways. Notably, kakkalide also demonstrated an improved capacity for enhancing insulin-mediated vasodilation in the rat aorta [92].

#### 4.2.5. Coumestrol

Coumestrol (C_15_H_8_O_5_) is a member of the coumestans, which is found in Campylotropis hirtella and Melilotus messanensis. Treatment with coumestrol improved retinopathy in diabetic rats and also improved HG-induced OS, inflammation, and cell apoptosis. Furthermore, human retinal endothelial cells (HRECs) were treated with short hairpin RNA-SIRT1 to explore the mechanisms, and the results indicate that coumestrol plays a protective role via the activation of SIRT1 [93].

### 4.3. Flavanols

The structural features of flavanols are the lack of a double bond at C2-C3, the lack of a carbonyl group at C4, and an additional hydroxyl group at C3 or C4. It is found in cassia nomame, beers, wine, tea, and cocoa [129].

#### 4.3.1. Dihydromyricetin

Dihydromyricetin (C_15_H_12_O_8_) is the hydrogenated derivative of myricetin, which is extracted from barnacles. To explore the mechanisms of dihydromyricetin on vascular dysfunction in diabetes, SIRT3 knockout mice were injected with STZ to construct diabetic models. The results indicate that dihydromyricetin alleviates endothelial dysfunction in diabetes via the inhibition of OS in a SIRT3-mediated way. Additionally, dihydromyricetin improved Ach-mediated endothelium-dependent relaxation [54].

#### 4.3.2. Silibinin

Silibinin (C_25_H_22_O_10_) is a kind of flavonolignan found in milk thistle. Silibinin has the potential to protect ECs from damage exposed to the HG environment via the recovery of autophagy, and it improved OS, cell migration, and viability. Furthermore, an intervention with the autophagy inhibitor 3-methyladenine, aim at discovering the effect of silibinin on HUVECs, suggested that silibinin initiated autophagy via the reduction in p62 levels and the enhancement in Beclin-1 and LC3 (microtubule-associated protein 1 light chain 3)-II/LC3-I levels [60].

#### 4.3.3. (–)-Epicatechin

(–)-Epicatechin (C_15_H_14_O_6_) is a natural product found in Visnea mocanera and Litsea rotundifolia. In HG-treated HUVECs, (–)-epicatechin improved endothelial dysfunction and reduced ROS by restoring the change in mitochondrial complexes induced by HG [17]. One study indicated that an increase in O-linked N-acetylglucosamine (O-GlcNAc) might participate in eNOS uncoupling in diabetes. (–)-Epicatechin reversed the increase in eNOS O-GlcNAc levels at Ser1177 induced by HG and restored mitochondrial biogenesis by increasing peroxisome proliferator-activated receptor γ coactivator 1α and SIRT1 levels [94].

Epigallocatechin-3-gallate (C_22_H_18_O_11_, EGCG) is a gallate ester based on (–)-epicatechin. In the aortic rings of diabetic rats induced by STZ, EGCG improved endothelial dysfunction by inhibiting PE and KCl-mediated endothelium-dependent contraction and enhancing Ach-mediated relaxation [95].

#### 4.3.4. Catechin

Catechin (C_15_H_14_O_6_) is a natural product found in Visnea mocanera and Salacia chinensis. Catechin prevented endothelial dysfunction by increasing Ach-mediated endothelium-dependent vasodilation in prediabetes, and it prevented OS via the inhibition of the overexpression of the NOX subunits p22^phox^ and p47^phox^ [130]. In addition, treatment with catechin hydrate improved endothelial function via the activation of the PI3K/eNOS/NO signaling pathways in diabetic rats [96].

#### 4.3.5. Proanthocyanidis

Proanthocyanidins are a type of polyphenol compound composed of flavanol monomers and their polymers. In the pulmonary artery rings of STZ-induced diabetic rats, a grapeseed proanthocyanidin extract (GSPE) improved OS and Ach and sodium nitroprusside-mediated vasodilation in diabetes [131,132]. In HUVECs, the GSPE inhibited the AGE-induced elevation in VCAM-1, RAGE, and ROS levels [133,134]. The GSPE improved mitochondrial function and reduced OS by increasing SIRT3 expression in HG-cultured EA.hy926 cells, as well as increasing SOD, CAT, and NO levels [135].

Procyanidin B2 (C_30_H_26_O_12_, PCB2) is a proanthocyanidin consisting of two (–)-epicatechins. It is found in Begonia fagifolia and Saraca asoca. PCB2 attenuated OS via the activation of Nrf2-related factors and protected cell proliferation and migration from HG damage [97]. PCB2 inhibited AGEs-induced apoptosis by regulating glycogen synthase kinase 3β phosphorylation [98]. PCB2 also protected cells from HG by inhibiting redoxosomes/NF-κB signaling and inhibiting the activation of NLRP3, caspase-1, and IL-1β in TR-iBRB2 cells [136].

### 4.4. Flavones

The features of flavone structures are an unsaturated carbon ring at C2-C3 and a ketone group at C4, while, compared to flavonols, lacking hydroxylation at C3 [137].

#### 4.4.1. Baicalein

Baicalein (C_15_H_10_O_5_) is a natural product that is characterized by three hydroxy groups at A5, A6, and A7. It is mainly found in Stachys annua and Stellera chamaejasme. Currently, some data have shown that baicalein protects endothelial function from OS via the Nrf2-mediated antioxidant system and MAPK signaling pathways in diabetic models. In vitro, EA. hy926 cells exposed to H_2_O_2_ showed a decrease in cell viability, NAD^+^/NADH, SOD activity, Nrf2, and heme oxygenase 1 (HO-1) expression, and these trends were reversed by baicalein. In vivo, baicalein protected arteries by improving eNOS expression and Nrf2-related antioxidant systems. Furthermore, the levels of pro-inflammatory factors were suppressed by baicalein [99]. Baicalein, an inhibitor of 12-lipoxygenase, inhibited STZ-induced erectile dysfunction via the activation of eNOS/NO/cGMP pathways, reduction in OS, and fibrosis. Furthermore, the results indicated that baicalein enhanced NO levels via the inhibition of p38/Arginase II/L-Arginine pathways; alleviated fibrosis via the inhibition of TGFβ1/p-Smad2/3/CTGF pathways; and attenuated OS via the reduction in NOX1 activity, ROS, and MDA levels and the increase in SOD activity [138]. In diabetic retinal complication models, treatment with baicalein decreased the overexpression of ICAM-1, VCAM-1, IL-6, and NOX2. Additionally, it also improved endothelial dysfunction via the inhibition of p-VEGF-receptor 2 [139].

Baicalin (C_21_H_18_O_11_) is a derivative of baicalein with 7-O-glucuronide, and it is the active ingredient of Scutellaria baicalensis. Treatment with baicalin protected chick embryos against cardiovascular malformation induced by HG, while the contents of SOD, GPx, and MDA were all attenuated after treatment with baicalin. The results indicate that baicalin might play an antioxidant role in chick embryos, but not by activating SOD and GPx. Moreover, baicalin improved p62-mediated autophagy [100].

Wogonoside (C_22_H_20_O_11_) is another ingredient in Scutellaria baicalensis, and in comparison to baicalin, its structure is characterized by an additional ether bond at A8 and a lack of a hydroxyl group at A6. In HRECs cultured with HG, wogonoside improved cell migration, proliferation, and membrane permeability by upregulating SIRT1 expression. It also inhibited HG-induced OS and inflammation by improving glutathione sulfotransferase activity and decreasing IL-1β and IL-6 levels [101].

Scutellarin (C_21_H_18_O_12_), compared to baicalin, has an attentional hydroxyl group at B4′. In HG-induced HUVECs, treatment with scutellarin inhibited EC apoptosis via a reduction in Bax/Bcl-2 and CytC expression and inhibited OS via a decrease in ROS levels and improved SOD2 expression; additionally, scutellarin improved mitochondrial autophagy via the activation of the PTEN-induced kinase 1/Parkin pathways [55]. It also protected HRECs from the attenuation of proliferation and migration exposed to HG and hypoxia conditions via the inhibition of ROS/hypoxia-inducible factor-1α (HIF-1α)/VEGF pathways [102].

#### 4.4.2. Luteolin

Luteolin (C_15_H_10_O_6_) is a naturally occurring flavonoid that is characterized by four hydroxy groups located at B3′, B4′, A5, and A7. It is found in Verbascum lychnitis and Carex fraseriana. In the aortic rings of diabetic rats induced by STZ, luteolin attenuated the HG-induced damage of endothelium-dependent relaxation via antioxidation and eNOS/NO pathways [103]. In Goto-Kakizaki rats, treatment with luteolin improved Ach-mediated vasodilation in arteries mounted with perivascular adipose tissue and reduced OS via an increase in MnSOD and GSH levels and a reduction in MDA levels and AR activity [140].

#### 4.4.3. Vaccarin

Vaccarin (C_32_H_38_O_19_) is a natural product found in Gypsophila vaccaria. The data showed that vaccarin had the potential to protect against endothelial dysfunction in diabetes. In diabetic mouse models induced by STZ and HFD, vaccarin restored the reduction in Ach-mediated endothelium-dependent vasorelaxation. In human microvascular endothelial cell-1 (HMEC-1) exposed to HG, vaccarin promoted p-eNOS, activated the AMPK pathway, and inhibited ROS production and elevation of microRNA (miRNA)-34a. Furthermore, an intervention with miRNA-34a and an AMPK inhibitor suggested that vaccarin improved endothelial dysfunction via the ROS/AMPK/miRNA-34a/eNOS pathways [104]. Additionally, vaccarin improved cell viability and inhibited cell apoptosis via the inhibition of histone deacetylase 1 in HG-cultured HMEC-1 cells [105].

#### 4.4.4. Apigenin

Apigenin (C_15_H_10_O_5_) is based on flavone replaced with three hydroxy groups at B4′, A5, and A7. The exposure of HUVECs to AGE environments induced inflammation and OS, and treatment with apigenin reduced ROS production and inflammation via the downregulation of RAGE, p-Erk1/2, NF-κB, MCP-1, and IL-6. Additionally, apigenin restored the levels of Nrf2 and related factors and HO-1. These observations indicated that apigenin might protect HUVECs against AGE-induced OS and inflammation via the inhibition of the RAGE/Erk/NF-κB pathways and the induction of Nrf2-related factors [141]. In HG-cultured HUVECs and HAECs, apigenin inhibited HG-induced PKCβII activation and ROS generation, increased Bcl-2 expression and the p-Akt/Akt ratio, and reduced Bax and caspase-3 expression and the NF-κB subunit p-p65/p65 ratio. Moreover, apigenin improved Ach-induced vasodilation and NO content in the aorta of Sprague Dawley rats exposed to an HG environment; furthermore, the observations indicated that apigenin might inhibit cell apoptosis and OS via the PKCβII-related ROS/caspase-3 pathways [109]. In the arteries of diabetic rats induced by STZ and HFD, apigenin also improved insulin and Ach-mediated vasodilation [106].

Vitexin is a combination of glycoside and apigenin. In HG-cultured HUVECs, treatment with vitexin reduced apoptosis via the activation of Wnt/b-catenin and increase in the Bcl-2/Bax ratio; additionally, vitexin attenuated OS via increases in SOD and Nrf2 activity [107].

### 4.5. Anthocyanins

Anthocyanins are natural pigments found in plants that are responsible for their vibrant colors. These pigments are stored in the vacuoles of plant cells. Anthocyanins are glycosides of anthocyanidins. They are water-soluble and remain unoxidized and unsaturated. These flavonoids are most abundant in flowers and fruits, where they contribute to pigmentation [142].

#### 4.5.1. Cyanidin

Cyanidin (C_15_H_11_O_6_^+^), which is flavylium replaced with hydroxy groups at C3, B3′, B4′, A5, and A7, is an anthocyanidin cation [8]. There are many derivatives of cyanidin in anthocyanin extracts of sour cherries, including cyanidin-3-rutinoside and cyanidin-3-o-glucoside (C_21_H_21_O_11_^+^, C3G). In HG-induced HUVECs, treatment with a sour cherry anthocyanin extract could inhibit excessive ROS generation; decrease TNF-α, IL-6, IL-8, and IL-1 levels; decrease the overexpression of ET-1 and endothelin-converting enzyme-1; and increase NOS expression. These results suggest that the sour cherry anthocyanin extract might inhibit HG-induced endothelial dysfunction via antioxidant, anti-inflammatory, and vasodilation enzyme activities [143]. The exposure of porcine aortic endothelial cells (PAECs) to glyLDL environments induced glyLDL-induced NOX activation, mitochondrial dysfunction, and a reduction in cell viability. Treatment with C3G could restore the viability of endothelial cells in PAECs under glyLDL conditions. Additionally, C3G reduced ROS production and NOX4 levels and restored the levels of the ETC complex I subunit NADH dehydrogenase subunit 1 and ETC complex III subunit CytB [18]. In HUVECS exposed to palmitic acid-induced toxicity and OS, pretreatment with C3G inhibited NF-κB-mediated inflammation and cell adhesion and restored TAC and GSH levels via the activation of Nrf2 [108].

#### 4.5.2. Malvidin

Malvidin (C_17_H_15_O_7_^+^) is delphinidin combined with methyl substituents at B3′ and B5′. It is a metabolite found in Saccharomyces cerevisiae. The predominant constituents of blueberry anthocyanin extracts (BAEs) include malvidin and its derivatives malvidin-3-glucoside and malvidin-3-galactoside. In HG-cultured HUVECs, BAE effectively reduced OS induced by HG by increasing the expression of SOD and HO-1 and the reduction in OS was achieved by inhibiting the generation of ROS and the overexpression of NOX4 and XO-1. BAE also restored vasodilation by increasing the levels of the NO, eNOS, and PPARγ and decreasing the levels of ACE and LDL. Furthermore, the findings demonstrate that BAE activates the PI3K/Akt pathways and blocks the PKCζ pathway. These results suggest that BAE protects endothelial function against HG-induced damage by exerting antioxidant effects and promoting vasodilation [144]. Furthermore, malvidin and malvidin-3-glucoside significantly protects HRECs against HG-induced inflammation via the downregulation of ICAM-1 and NF-κB [145].

### 4.6. Flavanones

Dihydroflavones, also referred to as flavanones, possess an oxidized, fully saturated carbon ring structure. Citrus fruits are abundant sources of flavanones, showcasing remarkable capabilities in neutralizing free radicals and exhibiting antioxidant properties [137].

#### 4.6.1. Naringenin

Naringenin (C_15_H_12_O_5_) is a natural compound that exists abundantly in citrus fruits [146]. The exposure of HRECs to HG led to inflammation, OS, and cell apoptosis. The observations indicated that the levels of TNF-α, IL-1β, ROS, Bax, and cleaved-caspase3 were increased, and eNOS, guanosine triphosphate cyclohydrolase-1 (GTPCH1), Bcl-2 expressions were inhibited. Co-treatment with naringenin inhibited this HG-induced damage. Furthermore, an intervention with siRNA-GTPCH1, aimed at exploring the mechanism and knockdown of GTPCH1, revealed a reverse protection of naringenin on HG-induced damage, and the results indicate that naringenin-mediated GTPCH1 upregulation is the key to protecting HRECs against HG-induced damage [147]. Compared to apigenin, naringenin showed better a protection of cell viability and anti-apoptosis and antioxidant capacities in HUVECs exposed to HG [109]. In the arteries of diabetic rats, naringenin significantly improved NO levels, PE-mediated contractions, and Ach or insulin-mediated relaxations [106,109].

Naringin is a naringenin derivative that is replaced with a 2-O-(α-L-rhamnopyranosyl)-β-D-glucopyranosyl moiety at A7. Naringin protected HUVECs against HG-induced cell viability and proliferation damages via the inhibition of ROS generation, improvement in p-Akt, and downregulation of CX3CL1 [110].

#### 4.6.2. Trans-Resveratrol

Trans-resveratrol (C_14_H_12_O_3_, t-RV) is the biologically active isomer of resveratrol that is found in grapevines. Some data have shown that t-RV protects against acute HG-induced cell damage. In HUVECs and rat aortic rings exposed to acute HG, the overproduction of ROS decreased cell viability, impaired Ach-mediated vasodilation was observed, and pretreatment with t-RV reduced these effects induced by acute HG [111].

#### 4.6.3. Didymin

Didymin (C_28_H_34_O_14_) is a naturally existing flavono-o-glycoside compound that is found in citrus fruits. Didymin was found to provide protection against cell death induced by HG via the reversal of the ROS/caspase-3/Bcl-2/MAPK signaling pathways and endothelial dysfunction via the NO/eNOS/ICAM/VCAM/NF-κB signaling pathways in HUVECs. Furthermore, it demonstrated a significant anti-inflammatory effect by inhibiting the overproduction of inflammatory factors induced by HG, including IL-1α, IL-8, IL-9, interferon-γ, and TNF-α [112].

#### 4.6.4. Liquiritin

Liquiritin (C_21_H_22_O_9_), which is liquiritigenin carrying a β-D-glucopyranosyl residue at B4′, is a flavanone glycoside. It is found in Polygonum aviculare and Artemisia capillaris. The exposure of HUVECs to AGE environments elicited endothelial apoptosis and OS, and treatment with liquiritin downregulated TGFβ1 and RAGE expression; decreased NF-κB activation, ROS generation, and MDA levels; and increased SOD activity. These observations suggested that liquiritin improved endothelial function via the RAGE/NF-κB pathways in HUVECs exposed to AGEs [113].

#### 4.6.5. Isoxanthohumol and Norkurarinone

Isoxanthohumol (C_21_H_22_O_5_, IXM) is a natural product found in Streptomyces, Humulus lupulus, and Sophora flavescens. Norkurarinone (C_25_H_28_O_6_, NKR) is a natural product found in Sophora stenophylla. IXM and NKR improved OS and cell autophagy via the activation of the PI3K/Akt/mTOR pathways and protected HRECs from HG and hypoxia-induced cell migration and angiogenesis by inhibiting the upregulation of HIF-1α and VEGF. Additionally, exposure to HG and hypoxic environments increased LC3-II/LC3-I, Beclin-1, and autophagy-related gene 5 mRNA levels and decreased p62 mRNA levels, and treatment with IXM and NKR reversed these trends [114].

### 4.7. Chalcones

Chalcones are compounds that are defined by their structure, consisting of two aryl moieties connected by an α, β-unsaturated carbonyl group. These compounds can be found in various sources, such as fruits, spices, teas, and soy [148].

#### Hydroxysafflor Yellow A

Hydroxysafflor yellow A (C_27_H_32_O_16_, HSYA) has a chalcone glycoside structure and is water-soluble. This compound is found in Carthamus tinctorius. In HG-cultured HUVECs, HSYA showed protection against OS via the inhibition of NOX4; reduced cell adhesion by inhibiting the overexpression of E-selectin, VCAM-1, and ICAM-1; and improved HG-induced hyperpermeability and cell apoptosis [80].

### 4.8. Deficiencies and Suggestions Regarding the above Study

Most of these studies lacked rescue experiments to validate them, which is not rigorous. Additionally, due to the detection of vasodilation, the research of the mechanisms is explored by in vitro experiments, which has some disadvantages. For example, an important effect of hyperglycemia is the degradation of Nrf2 due to the GSK-3beta-mediated instability of Nrf2. One effect of flavonoids may be the inhibition of GSK-3beta by activating the PI3K/Akt signaling pathway to inhibit the downregulation of basal Nrf2 levels [149,150]. But, in vitro, it is unclear whether the upregulation of Nrf2 levels is related to biological effects, because many phenolic compounds generate H_2_O_2_ in cell culture media [151], and many effects of flavonoids on cultured cells came from hydrogen peroxide that can activate Nrf2 to induce the upregulation of the antioxidant system, which is a confounding factor. It is recommended that more in vivo and rescue experiments are performed, and in addition to the validation of the targets, rescue experiments should also be performed to validate the relevant antioxidant enzymes in H_2_O_2_ production to ensure the rigor of the study.

## 5. Challenges and Perspectives Using Flavonoids and Their Derivatives to Treat Diabetic Complications

**Figure 2:**
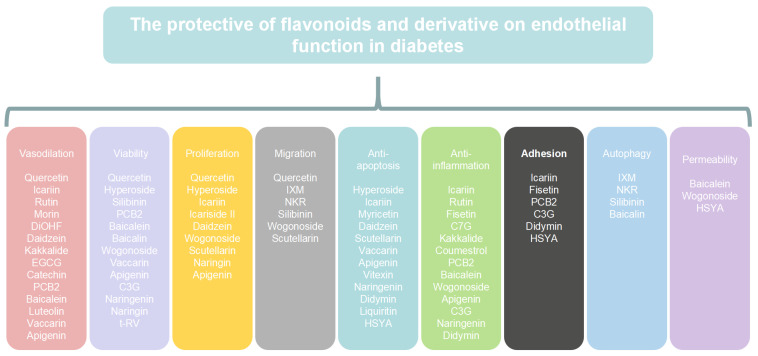
The protective action of flavonoids and their derivatives in endothelial function in diabetes. Abbreviations: DiOHF, 3′,4′-Dihydroxyflavonol; EGCG, epigallocatechin-3-gallate; PCB2, procyanidin B2; C3G, cyanidin-3-o-glucoside; t-RV, trans-resveratrol; IXM, isoxanthohumol; NKR, norkurarinone; HSYA, hydroxysafflor yellow A; C7G, calycosin-7-O-β-D-glucopyranoside.

Flavonoids and their derivatives have been shown to have the potential to mitigate the pathogenesis of diabetic complications. The antioxidant and anti-inflammatory properties of flavonoids contribute to the reduction in cell apoptosis, oxidative stress, inflammation, and cell adhesion. Additionally, flavonoids enhance vasodilation, mitochondrial function, cell viability, proliferation, and migration in individuals with diabetes (Figure 2). However, there are still many challenges to overcome in order to develop the applications of flavonoids.

Improving the protective effects of flavonoids has been under investigation. The data showed that the effect of flavonoids was closely associated with their structural characteristics. The antioxidant activity was positively correlated with hydroxyl groups and C2-C3 double bond, but negatively correlated with O-methyl groups and glycosyl groups. In contrast, the inhibition of p-Akt was positively correlated with C2-C3 double bond and hydroxyl group numbers in rings A and B, but negatively correlated with O-methyl groups, glycosyl groups, and hydroxyl groups in ring C [152]. These results provide a direction for synthesizing flavonoids. Furthermore, bioavailability is key to the effect of flavonoids. The data have shown that triglyceride-rich particles are the primary transporters of flavonoid metabolites, and diabetes inhibits the transportation of flavonoids via lipoproteins, leading to a reduction in their bioavailability in circulation [153]. Under physiological conditions, the low bioavailability of flavonoids is also an issue affecting their activity. The main factors that affect the flavonoids’ bioavailability include solubility affected by gastrointestinal pH, glycosidic bond structure, and intestinal permeation. Currently, there are still many unexplored areas regarding the intake style of flavonoids. Microencapsulation is a strategy that increases the absorption and bioavailability via the improvement in the residence in the intestine and particle endocytosis [154]. Graphene is a two-dimensional material composed of a single layer of carbon atoms arranged in a honeycomb lattice, and graphene derivatives could improve the biological activity by modifying the chemical structure of natural materials via surface conjugation and the change in oxidation state [155]. Chitooligosaccharide-functionalized graphene oxide could protect the stability of anthocyanins during intake and improve antioxidant activity [156]. A co-biopolymer of chitosan/carboxymethyl cellulose hydrogel modified by zinc oxide and graphene quantum dot nanoparticles improved the transportation of quercetin across the blood–brain barrier [157]. Additionally, the antioxidant activity of a blackcurrant anthocyanin extract was higher than that of anthocyanin alone, which may be related to the interaction of each component in the extract [158].

The dose is another important factor influencing the protection of flavonoids. The treatment of endothelial cells exposed to HG with apigenin or naringenin showed protective effects on cell viability, oxidative stress, and NO production. In general, compared to the low-dose group (3 μm), the high-dose group (30 μm) showed a better protective effect, while the low-dose group (3 μm) showed a better effect on the recovery of p-Akt/Akt [109]. The treatment with an anthocyanin-rich sour cherry extract reduced, in a concentration-dependent manner, HG-induced ROS in HUVECs, while the apoptotic cell ratio of the high-concentration group (50 ng/μL) was higher than that of the low-concentration group (1 ng/μL), which may contribute to the relatively increased mRNA expression of TNF-α, IL6, IL8, and IL1α [143]. Furthermore, the treatment with morin and naringenin resulted in an increase in ROS in nuclei isolated from rat livers [159]. Although the potential side effects of consuming excessive flavonoids are currently unclear, it is unlikely that the concentrations needed for most flavonoids to cause side effects can be obtained through dietary sources [8]. For example, the suggested range of quercetin supplement intake is commonly stated as 500–1000 mg/day, resulting in a dosage approximately 20-fold greater than what one might typically obtain from daily diet [160].

Vegetables and fruits have a low consumption of flavonoids. Additionally, the composition of vegetables and fruits encompasses not only flavonoids, but also a combination of secondary metabolites produced by plants. Different flavonoids extracted from plants have different effects. Anthocyanin compounds extracted from blueberries, which include malvidin and its derivatives, inhibit the overproduction of ROS induced by HG in HRECs. However, every constituent shows a different effect on the regulation of VEGF, Akt, ACE, eNOS, ICAM-1, and NF-κB [145]. Additionally, populations of different regions have different intake levels and consume different kinds of flavonoids [8]. Hence, it is difficult to construct a flavonoid dietary consumption guideline that is suitable for the whole world.

Currently, there is still a considerable need to explore unknown areas of flavonoids for the treatment of diabetes complications. From an epidemiological perspective, flavanols, flavones [161], and anthocyanidins could decrease the risk of diabetic nephropathy, and the consumption of a diet rich in flavonoids could reduce the risk of diabetic retinopathy and decrease the levels of HgbA1C and C-reactive protein [162]. Although these studies demonstrated the protective activity of flavonoid compounds on diabetes complications, there were still many limitations. For example, it is uncertain whether there is a direct causal relationship between dietary flavonoids and diabetic retinopathy. Flavonoids may improve diabetic retinopathy via the improvement in diabetes. Additionally, the presence of confounding factors generate bias and affect the results’ reliability. Mendelian randomization (MR) is a method based on genetics that uses genetic variation as an instrumental variable to assess causal relationships between exposure and outcome, and could promote the results’ reliability by avoiding confounding factors. Yuan et al. explored the causal relationships between plasma proteins and diabetic complications via MR [163]. Zheng et al. found that the treatment with glucagon-like peptide-1 receptor agonists decreased the risk of diabetic retinopathy [164]. With the increase in data, MR will be more widely used to explore the causal effects between flavonoids and other compounds.

There are many shortcomings in mechanistic research. There are studies that showed that the antioxidant activity of anthocyanins, in an in vitro digestion model, is higher than that of undigested cyanidin; the results indicate that the digestive process plays a significant role in enhancing the antioxidant activity [165,166]. This might be related to intestinal microorganisms that generate beneficial metabolites and regulate microbial composition [167]. Therefore, the simulation of the gastrointestinal environment could improve the accuracy of cellular experiments.

## 6. Conclusions

Flavonoids, which are abundantly found in plants, have predominantly protective effects against diabetes complications. Their antioxidant properties play a crucial role in protecting the endothelial function in diabetes. However, further research is needed to substantiate this promising avenue by exploring the mechanisms of action and to recommend intake programs of flavonoids and their derivatives.

## Figures and Tables

**Table 1:** The mechanisms of flavonoids and their derivatives in endothelial dysfunction in diabetes.

Flavonoid Subclass	Flavonoid Name	Molecular Formula	Mechanism	Model Used		References
				In Vivo	In Vitro	
Flavonol	Quercetin	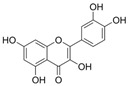	p-eNOS↑ p-AMPK↑ p-Akt↑		aorta of diabetic mice 10^−6^ M for 30 min	[41]
			MDA↓ ROS↓ p62↓ Beclin-1↑ LC3Ⅱ/Ⅰ↑ NO↑		HG-HUVECs 20 μM for 24/48/72 h	[58]
			SOD↑ CAT↑ MDA↓ protein carbonyls groups↓ iNOS↓	aorta of diabetic rats 30 mg/kg/day for 4 weeks gastric lavage		[39]
	Hyperoside	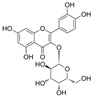	caspase-3↓ Bax↓ caspase-9↓ CytC↓ Bcl-2↑ SOD↑ MDA↓ ROS↓	retina of diabetic rats 20/50/100 mg/kg/day for 8 weeks gastric lavage	HG-RVECs 10 mg/mL for 72 h	[81]
	Icariin	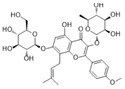	SOD↑ MDA↓ ROS↓ NOX4↓ p-p47^phox^↓ lactate dehydrogenase↓ caspase-3↓ Bcl-2/Bax↑ p65↓ NF-κB↓ IL-6↓ p-ERK/ERK↓ ICAM-1↓ VCAM-1↓ E-selectin↓		HG-HUVECs 50 μM for 72 h	[82]
	Icariside II	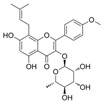	α-smooth muscle actin↑ collagen I/collagen III↑ TGFβ1↓ CTGF↓Smad2↓ nNOS↑ VEGF↑ eNOS↑ SOD↑ MDA↓ RAGE↓	penis of diabetic rats 1/5/10 mg/kg/day for 12 weeks, 5 mg/kg/day for 6 weeks gastric lavage		[83,84]
	Myricetin	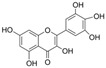	LPO↓ TAC↑ total thiol molecules↑ Bax/Bcl-2↓ cleaved caspase-3↓		HG-HUVECs 0.5/1 μM for 24 h	[85]
	Rutin	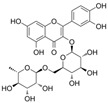	NOX4↓ ROS↓ TXNIP↓ NLRP3↓ IL-1β↓ caspase-1↓ NO↑	aorta of diabetic rats 35/70 mg/kg/day for 12 weeks	HG-HUVECs 10/30/100 μM for 0.5 h	[86]
	Fisetin	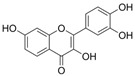	CAMs↓ MCP-1↓IL-8↓p65↓ H2O2↓	peritoneal exudates of mice 5.7/28.5 μg intravenous administration after 6h	HG-HUVECs 0.1/1/10/50μM for 6 h	[87]
			vWf↑ ICAM-1↓ eNOS↑ Erk-5↑		oxidized LDL-HUVECs 0.5 μM for 48 h	[88]
	Morin	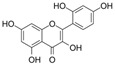	NO↑ p-eNOS↑ p-Akt↑		aorta of diabetic mice 10^−6^ M for 30 min	[41]
	DiOHF	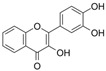	ROS↓ NOX2↓ eNOS↑	aorta of diabetic rats 1mg/kg/day for 7 days subcutaneous injection		[89]
Isoflavone	Daidzein	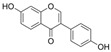	LPO↓ ROS↓ COX-2↓ CAT↑ SOD↑ GPx↑ iNOS↓ NF-κB↓		HG-HUVECs 0.02/0.04/0.1 μM for 20 h	[59]
	Puerarin	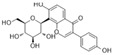	GSH↑ CAT↑ MDA↓ ROS↓ TXNIP↓ HMGB1↓ NLRP3↓ cleaved caspase-1↓		HG-mouse vascular endothelial Cells 1/10/25/50 μM for 24 h	[90]
	C7G	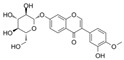	Bad↓ Bcl-2↑ Bax↓ MDA↓ SOD↑ IL-6↓ ICAM-1↓ MCP-1↓ TGFβ1↓ RAGE↓		AGEs-HUVECs 10^−7^/10^−8^/10^−9^ M for 24 h	[91]
	Kakkalide	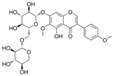	IL-6↓ TNF-α↓ ROS↓ p-JNK↓ p-p65↓ IκB kinase β↓ p-Akt↑ p-eNOS↑		palmitate–HUVECs palmitate–aorta of rats 0.1/1/10 μmol/L for 30 min	[92]
	Coumestrol	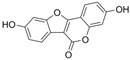	SIRT1↑IL-6↓ TNF-α↓ CytC in cytoplasm↓ CytC in mitochondria↑ SOD↑ ROS↓MDA↓ iNOS↓ NO↓ VEGF↓ cleaved caspase-3↓ C-reactive protein↓	retina of diabetic rats 10/50/100 mg/kg/day for 8 weeks subcutaneous injection	HG-HRECs	[93]
Flavanols	Dihydromyricetin	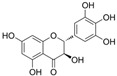	TAC↑ SIRT3↑ SOD2↑ GSH/GSSG↑ MDA↓ ROS↓	aorta of diabetic mice 250 mg/kg/day for 12 weeks gastric lavage		[54]
	Silibinin	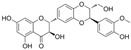	MDA↓ ROS↓ SOD↑ GSH↑ Beclin-1↑ p62↓ LC3-Ⅱ/LC3-Ⅰ↑ NO↓		HG-HUVECs 10 μM for 24/48/72 h	[60]
	(–)-Epicatechin	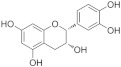	superoxide in mitochondrial↓ ETC complex V↓		HG-HUVECs 0.1/1 μM for 1 h	[17]
			eNOS O-GlcNAc↓ SIRT1↑ NO↑ p-eNOS↑		HG-HCACEs 100 μM for 48 h	[94]
	EGCG	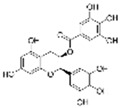	SOD↑ MDA↓	aorta of diabetic rats 25 mg/kg/day for 12 weeks gastric lavage		[95]
	Catechin	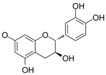	MDA↓ ROS↓	aorta of diabetic rats 50 mg/kg/day for 3 weeks gastric lavage		[96]
	PCB2	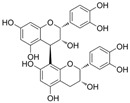	MDA↓ROS↓Nrf2↑CAT↑ NADPH dehydrogenase quinone 1↑		HG-EPCs 0.1/0.5/2.5 μmol/L for 6h	[97]
			cleaved caspase-3↓ lactadherin↓ ROS↓		AGE-HUVECs 2.5/5/10 μmol/l for 1 h	[98]
Flavones	Baicalein	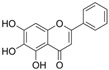	TNF-α↓p-p38↓ p-JNK↓ caspase-3↓ ROS↓ Nrf2↑ HO-1↑ SOD↑ eNOS↑ NAD+/NADH↑ MPO↓	aorta of HFD-Goto–Kakizaki rats 150 mg/kg/day for 4 weeks gastric lavage	H_2_O_2_-HUVECs 7.5/15 μg/mL for 1 h	[99]
	Baicalin	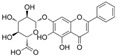	VEGF-receptor 2↑ MDA↓ cleaved caspase-3↓ p62↓	HG-induced chick embryos 6 μM for 48 h injection		[100]
	Wogonoside	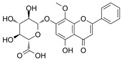	VEGF↓ HIF-1α↓ SIRT1↑ IL-1β↓ IL-6↓	retina of diabetic rats 30 mg/kg/day for 6 weeks	HG-HRECs 10/20/30/40 μmol/L	[101]
	Scutellarin	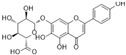	proliferating cell nuclear antigen↓ VEGF↓ HIF-1α↓ ROS↓ MDA↓ NOX↓		HG-HUVECs 1/10^−1^/ 10^−2^/10^−3^/ 10^−4^ μM for 48 h	[102]
	Luteolin	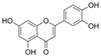	ROS↓ OH^·−^↓ SOD↑ NO↑ NOS↑	aorta of diabetic rats 10/50/100 mg/kg/day for 8 weeks		[103]
	Vaccarin	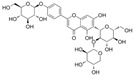	NO↑ p-eNOS↑ p-AMPK↑ ROS↓	aorta of diabetic mice 1 mg/kg/day for 4 weeks intraperitoneal injection	HG-HMEC-1 5 μM for 12 h	[104]
			Histone deacetylase 1↓ cleaved caspase-3↓ Bax/Bcl-2↓ SOD↑ CAT↑ GPx↑			[105]
	Apigenin	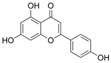	SOD↑ MDA↓ ICAM-1↓ NO↑ p-p65↓	artery of diabetic rats 50/100 mg/kg/day for 6 weeks gastric lavage		[106]
	Vitexin	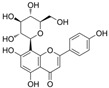	Bcl-2/Bax↑ Wnt/b-catenin↑ SOD↑ Nrf2↑ ROS↓ MDA↓		HG-HUVECs 15/30 μmol/L for 24 h	[107]
	C3G	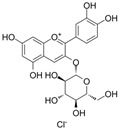	O_2_^·−^↓ NADH dehydrogenase↑ succinate cytochrome c reductase↑ CytC↑ NOX4↓ CytB↑ NADH dehydrogenase 1↑ cleaved caspase-3↓ Bcl-2↑		glyLDL-PAECs 30 μM for 12 h	[18]
			E-selectin↓ VCAM-1↓ leukocyte adhesion↓ ROS↓ Nrf2↑ NF-κB↓ HO-1↑ GSH↑ BTB and CNC homology 1↓ NADH quinone oxidoreductase 1↑		palmitic acid–HUVECs 20/40 μM for 24 h	[108]
Flavanones	Naringenin	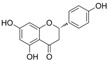	PKCβII↓ ROS↓ p-p65↓ p-Akt↑ Bcl-2↑ Bax↓ caspase-3↓ NO↑ p-eNOS↑		HG-HUVECs and HAECs 3/30 μM for 48 h	[109]
	Naringin	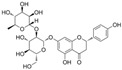	CX3XL1↓ p-Akt↑ ROS↓ NO↑		HG-HUVECs 50 μM for 48 h	[110]
	t-RV	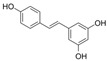	ROS↓		HG-HUVECs/rat aorta 0.1/1/10/ 100 μM for 3 h	[111]
	Didymin	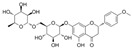	MDA↓ ROS↓ iNOS/eNOS↓ Bcl-2↑ Bax↓ caspase-3↓ ICAM-1↓ VCAM-1↓ NF-κB ↓ EGF↓ FGF2↓ CX3CL1↓ TNF-α↓ Interferon-α2↓ Interferon-γ↓ MCP-1↓ IL-1β↓ IL-1α↓ IL-2↓ IL-5↓ IL-6↓ IL-8↓ IL-15↓		HG-HUVECs 20 μM for 24 h	[112]
	Liquiritin	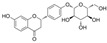	TGFβ1↓ RAGE↓ NF-κB↓ ROS↓ MDA↓ SOD ↑		AGEs-HUVECs 10^−6^/10^−7^/10^−8^ M for 48 h	[113]
	IXM	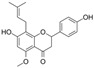	HIF-1α↓MDA↓ SOD↑ LC3-Ⅱ/LC3-Ⅰ↓ Beclin-1↓ p62↑ autophagy-related gene 5↓ p-PI3K↑ p-Akt↑ p-mTOR↑ VEGF↓		HG and hypoxia-HRECs high/ medium/ low dose for 24 h	[114]
	NKR	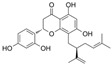				
Chalcones	HSYA	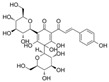	NOX4↓ ROS↓ H_2_O_2_↓ E-selectin↓ VCAM-1↓ ICAM-1↓ VEGF↓ Fibroblast growth factor↓		HG-HUVECs 50 μM for 24 h	[80]

Abbreviations: Red downward arrows, suppress; green upward arrows, enhance; p-eNOS, phosphorylation of eNOS; AMPK, phosphorylation of AMP-activated protein kinase; Akt, protein kinase B; MDA, malondialdehyde; ROS, reactive oxygen species; p62, sequestosome 1; LC3II, microtubule-associated protein 1A/1B-light chain 3-II; SOD, superoxide dismutase; CAT, catalase; iNOS, inducible nitric oxide synthase; CytC, cytochrome c; NOX4, NADPH oxidase 4; p47phox, phagocyte oxidase 47-kD subunit; Bcl-2, B-cell lymphoma 2; Bax, Bcl-2-associated X protein; NF-κB, nuclear factor-kappa B; ERK, extracellular signal-regulated kinase; ICAM-1, intercellular adhesion molecule 1; VCAM-1, vascular cell adhesion molecule-1; nNOS, neuronal nitric oxide synthase; VEGF, vascular endothelial growth factor; TGFβ1, transforming growth factor β1; CTGF, connective tissue growth factor; RAGE, receptor for advanced glycosylation end-products; LPO, lipid peroxidation; TAC, total antioxidant capacity; TXNIP, thioredoxin-interacting protein; NLRP3, nucleotide-binding domain-like receptor 3; MCP-1, monocyte chemotactic protein-1; COX-2, cyclooxygenase-2; GSH, glutathione; HMGB1, high mobility group box 1; JNK, c-Jun N-terminal kinase; SIRT1, Sirtuin 1; Nrf2, nuclear factor erythroid 2-related factor 2; HO-1, heme oxygenase 1; MPO, myeloperoxidase; HIF-1α, hypoxia-inducible factor 1-alpha; GPx, glutathione peroxidase; PKCβII, protein kinase C beta II; CX3XL1, fractalkine; EGF, epidermal growth factor; FGF2, fibroblast growth factor 2; IL-2, interleukin 2; PI3K, phosphatidylinositol 3-kinase; mTOR, mammalian target of rapamycin; DiOHF, 3′,4′-Dihydroxyflavonol; EGCG, epigallocatechin-3-gallate; PCB2, procyanidin B2; C3G, cyanidin-3-o-glucoside; t-RV, trans-resveratrol; IXM, isoxanthohumol; NKR, norkurarinone; HSYA, hydroxysafflor yellow A; C7G, caly-cosin-7-O-β-D-glucopyranoside.

## Data Availability

This article is a review and does not report original data. The data supporting this review are from previously reported studies and datasets, which have been cited.

## References

[1] International Diabetes Federation IDF Diabetes Atlas|Tenth Edition. https://diabetesatlas.org/.

[2] Ramanathan T., Skinner H. (2005). Coronary Blood Flow. Contin. Educ. Anaesth. Crit. Care Pain.

[3] Westein E., Hoefer T., Calkin A.C. (2017). Thrombosis in Diabetes: A Shear Flow Effect?. Clin. Sci. Lond. Engl. 1979.

[4] Xu S., Ilyas I., Little P.J., Li H., Kamato D., Zheng X., Luo S., Li Z., Liu P., Han J. (2021). Endothelial Dysfunction in Atherosclerotic Cardiovascular Diseases and Beyond: From Mechanism to Pharmacotherapies. Pharmacol. Rev..

[5] Incalza M.A., D’Oria R., Natalicchio A., Perrini S., Laviola L., Giorgino F. (2018). Oxidative Stress and Reactive Oxygen Species in Endothelial Dysfunction Associated with Cardiovascular and Metabolic Diseases. Vascul. Pharmacol..

[6] Shaito A., Aramouni K., Assaf R., Parenti A., Orekhov A., Yazbi A.E., Pintus G., Eid A.H. (2022). Oxidative Stress-Induced Endothelial Dysfunction in Cardiovascular Diseases. Front. Biosci. Landmark Ed..

[7] Mittal M., Siddiqui M.R., Tran K., Reddy S.P., Malik A.B. (2014). Reactive Oxygen Species in Inflammation and Tissue Injury. Antioxid. Redox Signal..

[8] Al-Ishaq R.K., Abotaleb M., Kubatka P., Kajo K., Büsselberg D. (2019). Flavonoids and Their Anti-Diabetic Effects: Cellular Mechanisms and Effects to Improve Blood Sugar Levels. Biomolecules.

[9] Gandhi G.R., Vasconcelos A.B.S., Wu D.-T., Li H.-B., Antony P.J., Li H., Geng F., Gurgel R.Q., Narain N., Gan R.-Y. (2020). Citrus Flavonoids as Promising Phytochemicals Targeting Diabetes and Related Complications: A Systematic Review of In Vitro and In Vivo Studies. Nutrients.

[10] Zibadi S., Rohdewald P.J., Park D., Watson R.R. (2008). Reduction of Cardiovascular Risk Factors in Subjects with Type 2 Diabetes by Pycnogenol Supplementation. Nutr. Res..

[11] Shen N., Wang T., Gan Q., Liu S., Wang L., Jin B. (2022). Plant Flavonoids: Classification, Distribution, Biosynthesis, and Antioxidant Activity. Food Chem..

[12] Tabit C.E., Chung W.B., Hamburg N.M., Vita J.A. (2010). Endothelial Dysfunction in Diabetes Mellitus: Molecular Mechanisms and Clinical Implications. Rev. Endocr. Metab. Disord..

[13] Shenouda S.M., Widlansky M.E., Chen K., Xu G., Holbrook M., Tabit C.E., Hamburg N.M., Frame A.A., Caiano T.L., Kluge M.A. (2011). Altered Mitochondrial Dynamics Contributes to Endothelial Dysfunction in Diabetes Mellitus. Circulation.

[14] Handy D.E., Loscalzo J. (2012). Redox Regulation of Mitochondrial Function. Antioxid. Redox Signal..

[15] Wang J., Toan S., Zhou H. (2020). New Insights into the Role of Mitochondria in Cardiac Microvascular Ischemia/Reperfusion Injury. Angiogenesis.

[16] Gerő D., Torregrossa R., Perry A., Waters A., Le-Trionnaire S., Whatmore J.L., Wood M., Whiteman M. (2016). The Novel Mitochondria-Targeted Hydrogen Sulfide (H2S) Donors AP123 and AP39 Protect against Hyperglycemic Injury in Microvascular Endothelial Cells in Vitro. Pharmacol. Res..

[17] Keller A., Hull S.E., Elajaili H., Johnston A., Knaub L.A., Chun J.H., Walker L., Nozik-Grayck E., Reusch J.E.B. (2020). (−)-Epicatechin Modulates Mitochondrial Redox in Vascular Cell Models of Oxidative Stress. Oxid. Med. Cell. Longev..

[18] Xie X., Zhao R., Shen G.X. (2012). Impact of Cyanidin-3-Glucoside on Glycated LDL-Induced NADPH Oxidase Activation, Mitochondrial Dysfunction and Cell Viability in Cultured Vascular Endothelial Cells. Int. J. Mol. Sci..

[19] Roy Chowdhury S.K., Sangle G.V., Xie X., Stelmack G.L., Halayko A.J., Shen G.X. (2010). Effects of Extensively Oxidized Low-Density Lipoprotein on Mitochondrial Function and Reactive Oxygen Species in Porcine Aortic Endothelial Cells. Am. J. Physiol. Endocrinol. Metab..

[20] Sangle G.V., Chowdhury S.K.R., Xie X., Stelmack G.L., Halayko A.J., Shen G.X. (2010). Impairment of Mitochondrial Respiratory Chain Activity in Aortic Endothelial Cells Induced by Glycated Low-Density Lipoprotein. Free Radic. Biol. Med..

[21] Faria A., Persaud S.J. (2017). Cardiac Oxidative Stress in Diabetes: Mechanisms and Therapeutic Potential. Pharmacol. Ther..

[22] Gorin Y., Block K. (2013). Nox as a Target for Diabetic Complications. Clin. Sci..

[23] Huang X., Sun M., Li D., Liu J., Guo H., Dong Y., Jiang L., Pan Q., Man Y., Wang S. (2011). Augmented NADPH Oxidase Activity and P22phox Expression in Monocytes Underlie Oxidative Stress of Patients with Type 2 Diabetes Mellitus. Diabetes Res. Clin. Pract..

[24] Taye A., Saad A.H., Kumar A.H., Morawietz H. (2010). Effect of Apocynin on NADPH Oxidase-Mediated Oxidative Stress-LOX-1-eNOS Pathway in Human Endothelial Cells Exposed to High Glucose. Eur. J. Pharmacol..

[25] Chen F., Qian L.-H., Deng B., Liu Z.-M., Zhao Y., Le Y.-Y. (2013). Resveratrol Protects Vascular Endothelial Cells from High Glucose-Induced Apoptosis through Inhibition of NADPH Oxidase Activation-Driven Oxidative Stress. CNS Neurosci. Ther..

[26] Williams C.R., Lu X., Sutliff R.L., Hart C.M. (2012). Rosiglitazone Attenuates NF-κB-Mediated Nox4 Upregulation in Hyperglycemia-Activated Endothelial Cells. Am. J. Physiol.-Cell Physiol..

[27] Ceolotto G., Gallo A., Papparella I., Franco L., Murphy E., Iori E., Pagnin E., Fadini G.P., Albiero M., Semplicini A. (2007). Rosiglitazone Reduces Glucose-Induced Oxidative Stress Mediated by NAD(P)H Oxidase via AMPK-Dependent Mechanism. Arterioscler. Thromb. Vasc. Biol..

[28] Christ M., Bauersachs J., Liebetrau C., Heck M., Günther A., Wehling M. (2002). Glucose Increases Endothelial-Dependent Superoxide Formation in Coronary Arteries by NAD(P)H Oxidase Activation: Attenuation by the 3-Hydroxy-3-Methylglutaryl Coenzyme A Reductase Inhibitor Atorvastatin. Diabetes.

[29] Quagliaro L., Piconi L., Assaloni R., Martinelli L., Motz E., Ceriello A. (2003). Intermittent High Glucose Enhances Apoptosis Related to Oxidative Stress in Human Umbilical Vein Endothelial Cells: The Role of Protein Kinase C and NAD(P)H-Oxidase Activation. Diabetes.

[30] Jha J.C., Dai A., Holterman C.E., Cooper M.E., Touyz R.M., Kennedy C.R., Jandeleit-Dahm K.A.M. (2019). Endothelial or Vascular Smooth Muscle Cell-Specific Expression of Human NOX5 Exacerbates Renal Inflammation, Fibrosis and Albuminuria in the Akita Mouse. Diabetologia.

[31] Chen F., Yu Y., Haigh S., Johnson J., Lucas R., Stepp D.W., Fulton D.J.R. (2014). Regulation of NADPH Oxidase 5 by Protein Kinase C Isoforms. PLoS ONE.

[32] Wendt M.C., Daiber A., Kleschyov A.L., Mülsch A., Sydow K., Schulz E., Chen K., Keaney J.F., Lassègue B., Walter U. (2005). Differential Effects of Diabetes on the Expression of the Gp91phox Homologues Nox1 and Nox4. Free Radic. Biol. Med..

[33] Youn J.Y., Gao L., Cai H. (2012). The P47phox- and NADPH Oxidase Organiser 1 (NOXO1)-Dependent Activation of NADPH Oxidase 1 (NOX1) Mediates Endothelial Nitric Oxide Synthase (eNOS) Uncoupling and Endothelial Dysfunction in a Streptozotocin-Induced Murine Model of Diabetes. Diabetologia.

[34] Sukumar P., Viswambharan H., Imrie H., Cubbon R.M., Yuldasheva N., Gage M., Galloway S., Skromna A., Kandavelu P., Santos C.X. (2013). Nox2 NADPH Oxidase Has a Critical Role in Insulin Resistance-Related Endothelial Cell Dysfunction. Diabetes.

[35] Wang L., Li X., Zhang Y., Huang Y., Zhang Y., Ma Q. (2019). Oxymatrine Ameliorates Diabetes-Induced Aortic Endothelial Dysfunction via the Regulation of eNOS and NOX4. J. Cell. Biochem..

[36] Förstermann U., Münzel T. (2006). Endothelial Nitric Oxide Synthase in Vascular Disease: From Marvel to Menace. Circulation.

[37] Hink U., Li H., Mollnau H., Oelze M., Matheis E., Hartmann M., Skatchkov M., Thaiss F., Stahl R.A., Warnholtz A. (2001). Mechanisms Underlying Endothelial Dysfunction in Diabetes Mellitus. Circ. Res..

[38] Daiber A., Chlopicki S. (2020). Revisiting Pharmacology of Oxidative Stress and Endothelial Dysfunction in Cardiovascular Disease: Evidence for Redox-Based Therapies. Free Radic. Biol. Med..

[39] Chis I., Coseriu A., Simedrea R., Oros A., Nagy A., Clichici S. (2015). In Vivo Effects of Quercetin in Association with Moderate Exercise Training in Improving Streptozotocin-Induced Aortic Tissue Injuries. Molecules.

[40] Taguchi K., Hida M., Hasegawa M., Matsumoto T., Kobayashi T. (2016). Dietary Polyphenol Morin Rescues Endothelial Dysfunction in a Diabetic Mouse Model by Activating the Akt/eNOS Pathway. Mol. Nutr. Food Res..

[41] Taguchi K., Tano I., Kaneko N., Matsumoto T., Kobayashi T. (2020). Plant Polyphenols Morin and Quercetin Rescue Nitric Oxide Production in Diabetic Mouse Aorta through Distinct Pathways. Biomed. Pharmacother..

[42] Pacher P., Nivorozhkin A., Szabó C. (2006). Therapeutic Effects of Xanthine Oxidase Inhibitors: Renaissance Half a Century after the Discovery of Allopurinol. Pharmacol. Rev..

[43] Hasan M., Fariha K.A., Barman Z., Mou A.D., Miah R., Habib A., Tuba H.R., Ali N. (2022). Assessment of the Relationship between Serum Xanthine Oxidase Levels and Type 2 Diabetes: A Cross-Sectional Study. Sci. Rep..

[44] Washio K., Kusunoki Y., Tsunoda T., Osugi K., Ohigashi M., Murase T., Nakamura T., Matsuo T., Konishi K., Katsuno T. (2020). Xanthine Oxidoreductase Activity Correlates with Vascular Endothelial Dysfunction in Patients with Type 1 Diabetes. Acta Diabetol..

[45] Okuyama T., Shirakawa J., Nakamura T., Murase T., Miyashita D., Inoue R., Kyohara M., Togashi Y., Terauchi Y. (2021). Association of the Plasma Xanthine Oxidoreductase Activity with the Metabolic Parameters and Vascular Complications in Patients with Type 2 Diabetes. Sci. Rep..

[46] Butler R., Morris A.D., Belch J.J., Hill A., Struthers A.D. (2000). Allopurinol Normalizes Endothelial Dysfunction in Type 2 Diabetics with Mild Hypertension. Hypertension.

[47] Desco M.-C., Asensi M., Márquez R., Martínez-Valls J., Vento M., Pallardó F.V., Sastre J., Viña J. (2002). Xanthine Oxidase Is Involved in Free Radical Production in Type 1 Diabetes: Protection by Allopurinol. Diabetes.

[48] Yang X.-D., Yang Y.-Y. (2022). Ferroptosis as a Novel Therapeutic Target for Diabetes and Its Complications. Front. Endocrinol..

[49] Luo E.-F., Li H.-X., Qin Y.-H., Qiao Y., Yan G.-L., Yao Y.-Y., Li L.-Q., Hou J.-T., Tang C.-C., Wang D. (2021). Role of Ferroptosis in the Process of Diabetes-Induced Endothelial Dysfunction. World J. Diabetes.

[50] Taguchi K., Okudaira K., Matsumoto T., Kobayashi T. (2023). Ginkgolide B Caused the Activation of the Akt/eNOS Pathway through the Antioxidant Effect of SOD1 in the Diabetic Aorta. Pflugers Arch..

[51] Juarez J.C., Manuia M., Burnett M.E., Betancourt O., Boivin B., Shaw D.E., Tonks N.K., Mazar A.P., Doñate F. (2008). Superoxide Dismutase 1 (SOD1) Is Essential for H2O2-Mediated Oxidation and Inactivation of Phosphatases in Growth Factor Signaling. Proc. Natl. Acad. Sci. USA.

[52] Morikawa K., Shimokawa H., Matoba T., Kubota H., Akaike T., Talukder M.A.H., Hatanaka M., Fujiki T., Maeda H., Takahashi S. (2003). Pivotal Role of Cu,Zn-Superoxide Dismutase in Endothelium-Dependent Hyperpolarization. J. Clin. Investig..

[53] Connor K.M., Subbaram S., Regan K.J., Nelson K.K., Mazurkiewicz J.E., Bartholomew P.J., Aplin A.E., Tai Y.-T., Aguirre-Ghiso J., Flores S.C. (2005). Mitochondrial H2O2 Regulates the Angiogenic Phenotype via PTEN Oxidation. J. Biol. Chem..

[54] Hua Y.-Y., Zhang Y., Gong W.-W., Ding Y., Shen J.-R., Li H., Chen Y., Meng G.-L. (2020). Dihydromyricetin Improves Endothelial Dysfunction in Diabetic Mice via Oxidative Stress Inhibition in a SIRT3-Dependent Manner. Int. J. Mol. Sci..

[55] Xi J., Rong Y., Zhao Z., Huang Y., Wang P., Luan H., Xing Y., Li S., Liao J., Dai Y. (2021). Scutellarin Ameliorates High Glucose-Induced Vascular Endothelial Cells Injury by Activating PINK1/Parkin-Mediated Mitophagy. J. Ethnopharmacol..

[56] Leff J.A., Oppegard M.A., Terada L.S., McCarty E.C., Repine J.E. (1991). Human Serum Catalase Decreases Endothelial Cell Injury from Hydrogen Peroxide. J. Appl. Physiol..

[57] Margis R., Dunand C., Teixeira F.K., Margis-Pinheiro M. (2008). Glutathione Peroxidase Family - an Evolutionary Overview. FEBS J..

[58] Rezabakhsh A., Rahbarghazi R., Malekinejad H., Fathi F., Montaseri A., Garjani A. (2019). Quercetin Alleviates High Glucose-Induced Damage on Human Umbilical Vein Endothelial Cells by Promoting Autophagy. Phytomedicine.

[59] Park M.H., Ju J.-W., Kim M., Han J.-S. (2016). The Protective Effect of Daidzein on High Glucose-Induced Oxidative Stress in Human Umbilical Vein Endothelial Cells. Z. Naturforschung C J. Biosci..

[60] Rezabakhsh A., Fathi F., Bagheri H.S., Malekinejad H., Montaseri A., Rahbarghazi R., Garjani A. (2018). Silibinin Protects Human Endothelial Cells from High Glucose-Induced Injury by Enhancing Autophagic Response. J. Cell. Biochem..

[61] Sharma P., Aggarwal K., Awasthi R., Kulkarni G.T., Sharma B. (2021). Behavioral and Biochemical Investigations to Explore the Efficacy of Quercetin and Folacin in Experimental Diabetes Induced Vascular Endothelium Dysfunction and Associated Dementia in Rats. J. Basic Clin. Physiol. Pharmacol..

[62] El Eter E., Al Masri A., Habib S., Al Zamil H., Al Hersi A., Al Hussein F., Al Omran M. (2014). Novel Links among Peroxiredoxins, Endothelial Dysfunction, and Severity of Atherosclerosis in Type 2 Diabetic Patients with Peripheral Atherosclerotic Disease. Cell Stress Chaperones.

[63] Li Y.R., Zhu H., Danelisen I. (2020). Role of Peroxiredoxins in Protecting Against Cardiovascular and Related Disorders. Cardiovasc. Toxicol..

[64] Nakamura T., Nakamura H., Hoshino T., Ueda S., Wada H., Yodoi J. (2005). Redox Regulation of Lung Inflammation by Thioredoxin. Antioxid. Redox Signal..

[65] Chong C.-R., Chan W.P.A., Nguyen T.H., Liu S., Procter N.E.K., Ngo D.T., Sverdlov A.L., Chirkov Y.Y., Horowitz J.D. (2014). Thioredoxin-Interacting Protein: Pathophysiology and Emerging Pharmacotherapeutics in Cardiovascular Disease and Diabetes. Cardiovasc. Drugs Ther..

[66] Haendeler J., Popp R., Goy C., Tischler V., Zeiher A.M., Dimmeler S. (2005). Cathepsin D and H2O2 Stimulate Degradation of Thioredoxin-1: Implication for Endothelial Cell Apoptosis. J. Biol. Chem..

[67] Li X., Kover K.L., Heruth D.P., Watkins D.J., Guo Y., Moore W.V., He L.G., Zang M., Clements M.A., Yan Y. (2017). Thioredoxin-Interacting Protein Promotes High-Glucose-Induced Macrovascular Endothelial Dysfunction. Biochem. Biophys. Res. Commun..

[68] Dunn L.L., Simpson P.J.L., Prosser H.C., Lecce L., Yuen G.S.C., Buckle A., Sieveking D.P., Vanags L.Z., Lim P.R., Chow R.W.Y. (2014). A Critical Role for Thioredoxin-Interacting Protein in Diabetes-Related Impairment of Angiogenesis. Diabetes.

[69] Lam Y.T., Tan R.P., Michael P., Yang N., Dunn L.L., Cooke J.P., Celermajer D.S., Wise S.G., Ng M.K.C. (2022). Endothelial Thioredoxin Interacting Protein (TXNIP) Modulates Endothelium-Dependent Vasorelaxation in Hyperglycemia. Microvasc. Res..

[70] Idris-Khodja N., Ouerd S., Mian M.O.R., Gornitsky J., Barhoumi T., Paradis P., Schiffrin E.L. (2016). Endothelin-1 Overexpression Exaggerates Diabetes-Induced Endothelial Dysfunction by Altering Oxidative Stress. Am. J. Hypertens..

[71] Ouerd S., Idris-Khodja N., Trindade M., Ferreira N.S., Berillo O., Coelho S.C., Neves M.F., Jandeleit-Dahm K.A., Paradis P., Schiffrin E.L. (2021). Endothelium-Restricted Endothelin-1 Overexpression in Type 1 Diabetes Worsens Atherosclerosis and Immune Cell Infiltration via NOX1. Cardiovasc. Res..

[72] Binjawhar D.N., Alhazmi A.T., Bin Jawhar W.N., MohammedSaeed W., Safi S.Z. (2023). Hyperglycemia-Induced Oxidative Stress and Epigenetic Regulation of ET-1 Gene in Endothelial Cells. Front. Genet..

[73] Issa E., Moss A.J., Fischer M., Kang M., Ahmed S., Farah H., Bate N., Giakomidi D., Brindle N.P. (2018). Development of an Orthogonal Tie2 Ligand Resistant to Inhibition by Ang2. Mol. Pharm..

[74] Akwii R.G., Sajib M.S., Zahra F.T., Mikelis C.M. (2019). Role of Angiopoietin-2 in Vascular Physiology and Pathophysiology. Cells.

[75] Puddu A., Sanguineti R., Maggi D., Nicolò M., Traverso C.E., Cordera R., Viviani G.L. (2019). Advanced Glycation End-Products and Hyperglycemia Increase Angiopoietin-2 Production by Impairing Angiopoietin-1-Tie-2 System. J. Diabetes Res..

[76] Peng X., Wang X., Fan M., Zhao J., Lin L., Liu J. (2020). Plasma Levels of von Willebrand Factor in Type 2 Diabetes Patients with and without Cardiovascular Diseases: A Meta-Analysis. Diabetes Metab. Res. Rev..

[77] Oggianu L., Lancellotti S., Pitocco D., Zaccardi F., Rizzo P., Martini F., Ghirlanda G., De Cristofaro R. (2013). The Oxidative Modification of von Willebrand Factor Is Associated with Thrombotic Angiopathies in Diabetes Mellitus. PLoS ONE.

[78] Kacso I.M., Potra A.R., Rusu A., Moldovan D., Rusu C.C., Kacso G., Hancu N.D., Muresan A., Bondor C.I. (2014). Relationship of Endothelial Cell Selective Adhesion Molecule to Markers of Oxidative Stress in Type 2 Diabetes. Scand. J. Clin. Lab. Investig..

[79] Inoue M., Ishida T., Yasuda T., Toh R., Hara T., Cangara H.M., Rikitake Y., Taira K., Sun L., Kundu R.K. (2010). Endothelial Cell-Selective Adhesion Molecule Modulates Atherosclerosis through Plaque Angiogenesis and Monocyte-Endothelial Interaction. Microvasc. Res..

[80] Chen S., Ma J., Zhu H., Deng S., Gu M., Qu S. (2019). Hydroxysafflor Yellow A Attenuates High Glucose-Induced Human Umbilical Vein Endothelial Cell Dysfunction. Hum. Exp. Toxicol..

[81] Wu W., Xie Z., Zhang Q., Ma Y., Bi X., Yang X., Li B., Chen J. (2020). Hyperoside Ameliorates Diabetic Retinopathy via Anti-Oxidation, Inhibiting Cell Damage and Apoptosis Induced by High Glucose. Front. Pharmacol..

[82] Sun S., Liu L., Tian X., Guo Y., Cao Y., Mei Y., Wang C. (2019). Icariin Attenuates High Glucose-Induced Apoptosis, Oxidative Stress, and Inflammation in Human Umbilical Venous Endothelial Cells. Planta Med..

[83] Zhou F., Xin H., Liu T., Li G.-Y., Gao Z.-Z., Liu J., Li W.-R., Cui W.-S., Bai G.-Y., Park N.C. (2012). Effects of Icariside II on Improving Erectile Function in Rats with Streptozotocin-Induced Diabetes. J. Androl..

[84] Wang L., Xu Y., Li H., Lei H., Guan R., Gao Z., Xin Z. (2015). Antioxidant Icariside II Combined with Insulin Restores Erectile Function in Streptozotocin-Induced Type 1 Diabetic Rats. J. Cell. Mol. Med..

[85] Aminzadeh A., Bashiri H. (2020). Myricetin Ameliorates High Glucose-Induced Endothelial Dysfunction in Human Umbilical Vein Endothelial Cells. Cell Biochem. Funct..

[86] Wang W., Wu Q.-H., Sui Y., Wang Y., Qiu X. (2017). Rutin Protects Endothelial Dysfunction by Disturbing Nox4 and ROS-Sensitive NLRP3 Inflammasome. Biomed. Pharmacother. Biomedecine Pharmacother..

[87] Kwak S., Ku S.-K., Bae J.-S. (2014). Fisetin Inhibits High-Glucose-Induced Vascular Inflammation in Vitro and in Vivo. Inflamm. Res..

[88] Patel R., Varghese J.F., Singh R.P., Yadav U.C.S. (2019). Induction of Endothelial Dysfunction by Oxidized Low-Density Lipoproteins via Downregulation of Erk-5/Mef2c/KLF2 Signaling: Amelioration by Fisetin. Biochimie.

[89] Ch L., Jl H., Ol W. (2011). 3’,4’-Dihydroxyflavonol Reduces Superoxide and Improves Nitric Oxide Function in Diabetic Rat Mesenteric Arteries. PLoS ONE.

[90] Lian D., Yuan H., Yin X., Wu Y., He R., Huang Y., Chen Y. (2019). Puerarin Inhibits Hyperglycemia-Induced Inter-Endothelial Junction through Suppressing Endothelial Nlrp3 Inflammasome Activation via ROS-Dependent Oxidative Pathway. Phytomedicine.

[91] Xu Y., Feng L., Wang S., Zhu Q., Lin J., Lou C., Xiang P., He B., Zheng Z., Tang D. (2011). Phytoestrogen Calycosin-7-O-β-D-Glucopyranoside Ameliorates Advanced Glycation End Products-Induced HUVEC Damage. J. Cell. Biochem..

[92] Zhang D., Gao X., Wang Q., Qin M., Liu K., Huang F., Liu B. (2013). Kakkalide Ameliorates Endothelial Insulin Resistance by Suppressing Reactive Oxygen Species-Associated Inflammation. J. Diabetes.

[93] Xu Y., Zhang Y., Liang H., Liu X. (2021). Coumestrol Mitigates Retinal Cell Inflammation, Apoptosis, and Oxidative Stress in a Rat Model of Diabetic Retinopathy via Activation of SIRT1. Aging.

[94] Ramírez-Sánchez I., Rodríguez A., Moreno-Ulloa A., Ceballos G., Villarreal F. (2016). (−)-Epicatechin-Induced Recovery of Mitochondria from Simulated Diabetes: Potential Role of Endothelial Nitric Oxide Synthase. Diab. Vasc. Dis. Res..

[95] Roghani M., Baluchnejadmojarad T. (2009). Chronic Epigallocatechin-Gallate Improves Aortic Reactivity of Diabetic Rats: Underlying Mechanisms. Vascul. Pharmacol..

[96] Bhardwaj P., Khanna D., Balakumar P. (2014). Catechin Averts Experimental Diabetes Mellitus-Induced Vascular Endothelial Structural and Functional Abnormalities. Cardiovasc. Toxicol..

[97] Fan J., Liu H., Wang J., Zeng J., Tan Y., Wang Y., Yu X., Li W., Wang P., Yang Z. (2021). Procyanidin B2 Improves Endothelial Progenitor Cell Function and Promotes Wound Healing in Diabetic Mice via Activating Nrf2. J. Cell. Mol. Med..

[98] Li B., Li X., Gao H., Zhang J., Cai Q., Cheng M., Lu M. (2011). Grape Seed Procyanidin B2 Inhibits Advanced Glycation End Product-Induced Endothelial Cell Apoptosis through Regulating GSK3β Phosphorylation. Cell Biol. Int..

[99] Wang C., Sun Y., Liu W., Liu Y., Afzal S., Grover J., Chang D., Münch G., Li C.G., Lin S. (2022). Protective Effect of the Curcumin-Baicalein Combination against Macrovascular Changes in Diabetic Angiopathy. Front. Endocrinol..

[100] Wang G., Liang J., Gao L.-R., Si Z.-P., Zhang X.-T., Liang G., Yan Y., Li K., Cheng X., Bao Y. (2018). Baicalin Administration Attenuates Hyperglycemia-Induced Malformation of Cardiovascular System. Cell Death Dis..

[101] Shao X., Yu J., Ni W. (2022). Wogonoside alleviates high glucose-induced dysfunction of retinal microvascular endothelial cells and diabetic retinopathy in rats by up-regulating SIRT1. Nan Fang Yi Ke Da Xue Xue Bao.

[102] Wang D., Wang L., Gu J., Yang H., Liu N., Lin Y., Li X., Shao C. (2014). Scutellarin Inhibits High Glucose-Induced and Hypoxia-Mimetic Agent-Induced Angiogenic Effects in Human Retinal Endothelial Cells through Reactive Oxygen Species/Hypoxia-Inducible Factor-1α/Vascular Endothelial Growth Factor Pathway. J. Cardiovasc. Pharmacol..

[103] Qian L.-B., Wang H.-P., Chen Y., Chen F.-X., Ma Y.-Y., Bruce I.C., Xia Q. (2010). Luteolin Reduces High Glucose-Mediated Impairment of Endothelium-Dependent Relaxation in Rat Aorta by Reducing Oxidative Stress. Pharmacol. Res..

[104] Xu F., Liu Y., Zhu X., Li S., Shi X., Li Z., Ai M., Sun J., Hou B., Cai W. (2019). Protective Effects and Mechanisms of Vaccarin on Vascular Endothelial Dysfunction in Diabetic Angiopathy. Int. J. Mol. Sci..

[105] Zhu X., Lei Y., Tan F., Gong L., Gong H., Yang W., Chen T., Zhang Z., Cai W., Hou B. (2018). Vaccarin Protects Human Microvascular Endothelial Cells from Apoptosis via Attenuation of HDAC1 and Oxidative Stress. Eur. J. Pharmacol..

[106] Ren B., Qin W., Wu F., Wang S., Pan C., Wang L., Zeng B., Ma S., Liang J. (2016). Apigenin and Naringenin Regulate Glucose and Lipid Metabolism, and Ameliorate Vascular Dysfunction in Type 2 Diabetic Rats. Eur. J. Pharmacol..

[107] Zhang S., Jin S., Zhang S., Li Y.-Y., Wang H., Chen Y., Lu H. (2022). Vitexin Protects against High Glucose-Induced Endothelial Cell Apoptosis and Oxidative Stress via Wnt/β-Catenin and Nrf2 Signalling Pathway. Arch. Physiol. Biochem..

[108] Fratantonio D., Speciale A., Ferrari D., Cristani M., Saija A., Cimino F. (2015). Palmitate-Induced Endothelial Dysfunction Is Attenuated by Cyanidin-3-O-Glucoside through Modulation of Nrf2/Bach1 and NF-κB Pathways. Toxicol. Lett..

[109] Qin W., Ren B., Wang S., Liang S., He B., Shi X., Wang L., Liang J., Wu F. (2016). Apigenin and Naringenin Ameliorate PKCβII-Associated Endothelial Dysfunction via Regulating ROS/Caspase-3 and NO Pathway in Endothelial Cells Exposed to High Glucose. Vascul. Pharmacol..

[110] Li G., Xu Y., Sheng X., Liu H., Guo J., Wang J., Zhong Q., Jiang H., Zheng C., Tan M. (2017). Naringin Protects Against High Glucose-Induced Human Endothelial Cell Injury Via Antioxidation and CX3CL1 Downregulation. Cell. Physiol. Biochem..

[111] Guzmán L., Balada C., Flores G., Álvarez R., Knox M., Vinet R., Martínez J.L. (2018). T-Resveratrol Protects against Acute High Glucose Damage in Endothelial Cells. Plant Foods Hum. Nutr..

[112] Shukla K., Sonowal H., Saxena A., Ramana K.V. (2018). Didymin Prevents Hyperglycemia-Induced Human Umbilical Endothelial Cells Dysfunction and Death. Biochem. Pharmacol..

[113] Zhang X., Song Y., Han X., Feng L., Wang R., Zhang M., Zhu M., Jia X., Hu S. (2013). Liquiritin Attenuates Advanced Glycation End Products-Induced Endothelial Dysfunction via RAGE/NF-κB Pathway in Human Umbilical Vein Endothelial Cells. Mol. Cell. Biochem..

[114] Zhao K., Li Y., Qiu Y., Huang R., Lin M., Chen L., Liu Y. (2022). Norkurarinone and Isoxanthohumol Inhibit High Glucose and Hypoxia-Induced Angiogenesis via Improving Oxidative Stress and Regulating Autophagy in Human Retinal Microvascular Endothelial Cells. Biochem. Biophys. Res. Commun..

[115] Kozłowska A., Szostak-Węgierek D. (2022). Targeting Cardiovascular Diseases by Flavonols: An Update. Nutrients.

[116] Anand David A., Arulmoli R., Parasuraman S. (2016). Overviews of Biological Importance of Quercetin: A Bioactive Flavonoid. Pharmacogn. Rev..

[117] Boesten D.M.P.H.J., von Ungern-Sternberg S.N.I., den Hartog G.J.M., Bast A. (2015). Protective Pleiotropic Effect of Flavonoids on NAD^+^ Levels in Endothelial Cells Exposed to High Glucose. Oxid. Med. Cell. Longev..

[118] Tian R., Jin Z., Zhou L., Zeng X.-P., Lu N. (2021). Quercetin Attenuated Myeloperoxidase-Dependent HOCl Generation and Endothelial Dysfunction in Diabetic Vasculature. J. Agric. Food Chem..

[119] Zhao L.-R., Du Y.-J., Chen L., Liu Z.-G., Pan Y.-H., Liu J.-F., Liu B. (2014). Quercetin Protects against High Glucose-Induced Damage in Bone Marrow-Derived Endothelial Progenitor Cells. Int. J. Mol. Med..

[120] Wang X., Yao W., Pan Z., Dong J., Liu S., Ding X. (2022). Protective effects and mechanism of icariin against vascular function in diabetic mice. China Pharm. Univ..

[121] Tang Y., Jacobi A., Vater C., Zou L., Zou X., Stiehler M. (2015). Icariin Promotes Angiogenic Differentiation and Prevents Oxidative Stress-Induced Autophagy in Endothelial Progenitor Cells. Stem Cells Dayt. Ohio.

[122] Li H., Xu Y., Guan R., Matheu M., Lei H., Tian W., Gao Z., Lin G., Guo Y., Xin Z. (2015). Icariside II Prevents High-Glucose-Induced Injury on Human Cavernous Endothelial Cells through Akt-eNOS Signaling Pathway. Andrology.

[123] Tian W., Lei H., Guan R., Xu Y., Li H., Wang L., Yang B., Gao Z., Xin Z. (2015). Icariside II Ameliorates Diabetic Nephropathy in Streptozotocin-Induced Diabetic Rats. Drug Des. Devel. Ther..

[124] Wang X., Zhao X., Feng T., Jin G., Li Z. (2016). Rutin Prevents High Glucose-Induced Renal Glomerular Endothelial Hyperpermeability by Inhibiting the ROS/Rhoa/ROCK Signaling Pathway. Planta Med..

[125] Woodman O.L., Malakul W. (2009). 3’,4’-Dihydroxyflavonol Prevents Diabetes-Induced Endothelial Dysfunction in Rat Aorta. Life Sci..

[126] Leo C.-H., Hart J.L., Woodman O.L. (2011). 3’,4’-Dihydroxyflavonol Restores Endothelium-Dependent Relaxation in Small Mesenteric Artery from Rats with Type 1 and Type 2 Diabetes. Eur. J. Pharmacol..

[127] Roghani M., Vaez Mahdavi M.-R., Jalali-Nadoushan M.-R., Baluchnejadmojarad T., Naderi G., Roghani-Dehkordi F., Taghi Joghataei M., Kord M. (2013). Chronic Administration of Daidzein, a Soybean Isoflavone, Improves Endothelial Dysfunction and Attenuates Oxidative Stress in Streptozotocin-Induced Diabetic Rats. Phytother. Res. PTR.

[128] Xu S.-Z., Zhong W., Ghavideldarestani M., Saurabh R., Lindow S.W., Atkin S.L. (2009). Multiple Mechanisms of Soy Isoflavones against Oxidative Stress-Induced Endothelium Injury. Free Radic. Biol. Med..

[129] Luo Y., Jian Y., Liu Y., Jiang S., Muhammad D., Wang W. (2022). Flavanols from Nature: A Phytochemistry and Biological Activity Review. Molecules.

[130] Ihm S.-H., Lee J.-O., Kim S.-J., Seung K.-B., Schini-Kerth V.B., Chang K., Oak M.-H. (2009). Catechin Prevents Endothelial Dysfunction in the Prediabetic Stage of OLETF Rats by Reducing Vascular NADPH Oxidase Activity and Expression. Atherosclerosis.

[131] Pinna C., Morazzoni P., Sala A. (2017). Proanthocyanidins from Vitis Vinifera Inhibit Oxidative Stress-Induced Vascular Impairment in Pulmonary Arteries from Diabetic Rats. Phytomedicine.

[132] Okudan N., Barışkaner H., Gökbel H., Sahin A.S., Belviranlı M., Baysal H. (2011). The Effect of Supplementation of Grape Seed Proanthocyanidin Extract on Vascular Dysfunction in Experimental Diabetes. J. Med. Food.

[133] Zhang F.-L., Gao H.-Q., Wu J.-M., Ma Y.-B., You B.-A., Li B.-Y., Xuan J.-H. (2006). Selective Inhibition by Grape Seed Proanthocyanidin Extracts of Cell Adhesion Molecule Expression Induced by Advanced Glycation End Products in Endothelial Cells. J. Cardiovasc. Pharmacol..

[134] Zhang F.-L., Gao H.-Q., Shen L. (2007). Inhibitory Effect of GSPE on RAGE Expression Induced by Advanced Glycation End Products in Endothelial Cells. J. Cardiovasc. Pharmacol..

[135] Cerbaro A.F., Rodrigues V.S.B., Rigotti M., Branco C.S., Rech G., de Oliveira D.L., Salvador M. (2020). Grape Seed Proanthocyanidins Improves Mitochondrial Function and Reduces Oxidative Stress through an Increase in Sirtuin 3 Expression in EA. Hy926 Cells in High Glucose Condition. Mol. Biol. Rep..

[136] Zou W., Lu Q., Zhu X., Pan Y., Xu Q., Wang K. (2022). Procyanidin B2 Protects TR-iBRB2 Cells Against Hyperglycemia Stress by Attenuating Oxidative Stress and Inflammasome Activation via Regulation of Redoxosomes/NF-kB Signaling. Curr. Mol. Med..

[137] Panche A.N., Diwan A.D., Chandra S.R. (2016). Flavonoids: An Overview. J. Nutr. Sci..

[138] Chen Y., Zhou B., Yu Z., Yuan P., Sun T., Gong J., Zhang Y., Wang T., Wang S., Liu K. (2020). Baicalein Alleviates Erectile Dysfunction Associated With Streptozotocin-Induced Type I Diabetes by Ameliorating Endothelial Nitric Oxide Synthase Dysfunction, Inhibiting Oxidative Stress and Fibrosis. J. Sex. Med..

[139] Othman A., Ahmad S., Megyerdi S., Mussell R., Choksi K., Maddipati K.R., Elmarakby A., Rizk N., Al-Shabrawey M. (2013). 12/15-Lipoxygenase-Derived Lipid Metabolites Induce Retinal Endothelial Cell Barrier Dysfunction: Contribution of NADPH Oxidase. PLoS ONE.

[140] Queiroz M., Leandro A., Azul L., Figueirinha A., Seiça R., Sena C.M. (2021). Luteolin Improves Perivascular Adipose Tissue Profile and Vascular Dysfunction in Goto-Kakizaki Rats. Int. J. Mol. Sci..

[141] Zhou Q., Cheng K.-W., Gong J., Li E.T.S., Wang M. (2019). Apigenin and Its Methylglyoxal-Adduct Inhibit Advanced Glycation End Products-Induced Oxidative Stress and Inflammation in Endothelial Cells. Biochem. Pharmacol..

[142] Putta S., Yarla N.S., Kumar K E., Lakkappa D.B., Kamal M.A., Scotti L., Scotti M.T., Ashraf G.M., Rao B.S.B., D S.K. (2018). Preventive and Therapeutic Potentials of Anthocyanins in Diabetes and Associated Complications. Curr. Med. Chem..

[143] Markovics A., Biró A., Kun-Nemes A., Fazekas M.É., Rácz A.A., Paholcsek M., Lukács J., Stündl L., Remenyik J. (2020). Effect of Anthocyanin-Rich Extract of Sour Cherry for Hyperglycemia-Induced Inflammatory Response and Impaired Endothelium-Dependent Vasodilation. Nutrients.

[144] Huang W., Hutabarat R.P., Chai Z., Zheng T., Zhang W., Li D. (2020). Antioxidant Blueberry Anthocyanins Induce Vasodilation via PI3K/Akt Signaling Pathway in High-Glucose-Induced Human Umbilical Vein Endothelial Cells. Int. J. Mol. Sci..

[145] Huang W., Yan Z., Li D., Ma Y., Zhou J., Sui Z. (2018). Antioxidant and Anti-Inflammatory Effects of Blueberry Anthocyanins on High Glucose-Induced Human Retinal Capillary Endothelial Cells. Oxid. Med. Cell. Longev..

[146] Hasanein P., Fazeli F. (2014). Role of Naringenin in Protection against Diabetic Hyperalgesia and Tactile Allodynia in Male Wistar Rats. J. Physiol. Biochem..

[147] Xue B., Wang Y. (2022). Naringenin Upregulates GTPCH1/eNOS to Ameliorate High Glucose-Induced Retinal Endothelial Cell Injury. Exp. Ther. Med..

[148] Elkanzi N.A.A., Hrichi H., Alolayan R.A., Derafa W., Zahou F.M., Bakr R.B. (2022). Synthesis of Chalcones Derivatives and Their Biological Activities: A Review. ACS Omega.

[149] Li J., Wang T., Liu P., Yang F., Wang X., Zheng W., Sun W. (2021). Hesperetin Ameliorates Hepatic Oxidative Stress and Inflammation via the PI3K/AKT-Nrf2-ARE Pathway in Oleic Acid-Induced HepG2 Cells and a Rat Model of High-Fat Diet-Induced NAFLD. Food Funct..

[150] Zhou Z., Li H., Bai S., Xu Z., Jiao Y. (2022). Loss of Serine/Threonine Protein Kinase 25 in Retinal Ganglion Cells Ameliorates High Glucose-Elicited Damage through Regulation of the AKT-GSK-3β/Nrf2 Pathway. Biochem. Biophys. Res. Commun..

[151] Long L.H., Hoi A., Halliwell B. (2010). Instability of, and Generation of Hydrogen Peroxide by, Phenolic Compounds in Cell Culture Media. Arch. Biochem. Biophys..

[152] Dirimanov S., Högger P. (2019). Screening of Inhibitory Effects of Polyphenols on Akt-Phosphorylation in Endothelial Cells and Determination of Structure-Activity Features. Biomolecules.

[153] Reis A., Rocha S., Dias I.H., Costa R., Soares R., Sánchez-Quesada J.L., Perez A., de Freitas V. (2023). Type 2 Diabetes Mellitus Alters the Cargo of (Poly)Phenol Metabolome and the Oxidative Status in Circulating Lipoproteins. Redox Biol..

[154] Annunziata G., Jiménez-García M., Capó X., Moranta D., Arnone A., Tenore G.C., Sureda A., Tejada S. (2020). Microencapsulation as a Tool to Counteract the Typical Low Bioavailability of Polyphenols in the Management of Diabetes. Food Chem. Toxicol..

[155] Lakshmanan R., Maulik N. (2018). Graphene-Based Drug Delivery Systems in Tissue Engineering and Nanomedicine. Can. J. Physiol. Pharmacol..

[156] Xue B., Wang Y., Tian J., Zhang W., Zang Z., Cui H., Zhang Y., Jiang Q., Li B., Hai Liu R. (2022). Effects of Chitooligosaccharide-Functionalized Graphene Oxide on Stability, Simulated Digestion, and Antioxidant Activity of Blueberry Anthocyanins. Food Chem..

[157] Ostovar S., Pourmadadi M., Zaker M.A. (2023). Co-Biopolymer of Chitosan/Carboxymethyl Cellulose Hydrogel Improved by Zinc Oxide and Graphene Quantum Dots Nanoparticles as pH-Sensitive Nanocomposite for Quercetin Delivery to Brain Cancer Treatment. Int. J. Biol. Macromol..

[158] Nielsen I.L.F., Dragsted L.O., Ravn-Haren G., Freese R., Rasmussen S.E. (2003). Absorption and Excretion of Black Currant Anthocyanins in Humans and Watanabe Heritable Hyperlipidemic Rabbits. J. Agric. Food Chem..

[159] Skibola C.F., Smith M.T. (2000). Potential Health Impacts of Excessive Flavonoid Intake. Free Radic. Biol. Med..

[160] Sahu S.C., Gray G.C. (1993). Interactions of Flavonoids, Trace Metals, and Oxygen: Nuclear DNA Damage and Lipid Peroxidation Induced by Myricetin. Cancer Lett..

[161] Liu F., Nie J., Deng M.-G., Yang H., Feng Q., Yang Y., Li X., Li X., Yang X., Li W. (2023). Dietary Flavonoid Intake Is Associated with a Lower Risk of Diabetic Nephropathy in US Adults: Data from NHANES 2007-2008, 2009-2010, and 2017-2018. Food Funct..

[162] Mahoney S.E., Loprinzi P.D. (2014). Influence of Flavonoid-Rich Fruit and Vegetable Intake on Diabetic Retinopathy and Diabetes-Related Biomarkers. J. Diabetes Complicat..

[163] Yuan S., Xu F., Li X., Chen J., Zheng J., Mantzoros C.S., Larsson S.C. (2023). Plasma Proteins and Onset of Type 2 Diabetes and Diabetic Complications: Proteome-Wide Mendelian Randomization and Colocalization Analyses. Cell Rep. Med..

[164] Zheng D., Li N., Hou R., Zhang X., Wu L., Sundquist J., Sundquist K., Ji J. (2023). Glucagon-like Peptide-1 Receptor Agonists and Diabetic Retinopathy: Nationwide Cohort and Mendelian Randomization Studies. BMC Med..

[165] David L., Danciu V., Moldovan B., Filip A. (2019). Effects of In Vitro Gastrointestinal Digestion on the Antioxidant Capacity and Anthocyanin Content of Cornelian Cherry Fruit Extract. Antioxidants.

[166] McDougall G.J., Fyffe S., Dobson P., Stewart D. (2005). Anthocyanins from Red Wine--Their Stability under Simulated Gastrointestinal Digestion. Phytochemistry.

[167] Carrera-Quintanar L., López Roa R.I., Quintero-Fabián S., Sánchez-Sánchez M.A., Vizmanos B., Ortuño-Sahagún D. (2018). Phytochemicals That Influence Gut Microbiota as Prophylactics and for the Treatment of Obesity and Inflammatory Diseases. Mediators Inflamm..

